# The *Rhizobium tropici* CIAT 899 NodD2 protein regulates the production of Nod factors under salt stress in a flavonoid-independent manner

**DOI:** 10.1038/srep46712

**Published:** 2017-05-10

**Authors:** Pablo del Cerro, Francisco Pérez-Montaño, Antonio Gil-Serrano, Francisco Javier López-Baena, Manuel Megías, Mariangela Hungria, Francisco Javier Ollero

**Affiliations:** 1 Departamento de Microbiología, Facultad de Biología, Universidad de Sevilla, Sevilla, Spain; 2 Departamento de Química Orgánica, Facultad de Química, Universidad de Sevilla, Sevilla, Spain; 3 Embrapa Soja, Londrina, Paraná, Brazil

## Abstract

In the symbiotic associations between rhizobia and legumes, NodD promotes the expression of the nodulation genes in the presence of appropriate flavonoids. This set of genes is implied in the synthesis of Nodulation factors, which are responsible for launching the nodulation process. *Rhizobium tropici* CIAT 899 is the most successful symbiont of *Phaseolus vulgaris* and can nodulate a variety of legumes. This strain produces Nodulation factors under abiotic stress such as acidity or high concentration of salt. Genome sequencing of CIAT 899 allowed the identification of five *nodD* genes. Whereas NodD1 is essential to nodulate *Leucaena leucocephala, Lotus japonicus* and *Macroptilium atropurpureum*, symbiosis with *P. vulgaris* and *Lotus burtii* decreased the nodule number but did not abolish the symbiotic process when NodD1 is absent. Nodulation factor synthesis under salt stress is not regulated by NodD1. Here we confirmed that NodD2 is responsible for the activation of the CIAT 899 symbiotic genes under salt stress. We have demonstrated that NodD1 and NodD2 control the synthesis of the Nod factor necessary for a successful symbiosis with *P. vulgaris* and *L. burtii*. This is the first time that NodD is directly implied in the activation of the symbiotic genes under an abiotic stress.

Flavonoids are phenylpropanoid metabolites exuded from plant roots, often in response to elicitors generated by microorganisms present in the rhizosphere^1^. These molecules are antimicrobial compounds that inhibit growth of bacterial and fungal root pathogens^2^. However, in rhizobia, flavonoids act as bacterial growth promoters, chemoattractants, inducers of nodulation genes, determinants of host specificity, and regulators of phytoalexin resistance^3^. Rhizobia establish symbiotic associations with leguminous plants, evolving fine tuning events that culminate in the formation of nodules and the fixation of the atmospheric nitrogen (N_2_) into ammonia^4^. Thus, rhizobia have managed to reorient the biotic stress caused by flavonoids towards the symbiotic process. This adaptation is mediated by the NodD protein, a LysR-type transcriptional regulator constitutively expressed that promotes the expression of the nodulation genes (*nod* genes) in the presence of appropriate flavonoids by binding to conserved promoter regions (*nod* boxes, NB) located upstream of these genes^5^,^6^,^7^,^8^,^9^. The *nod* genes, located in the symbiotic plasmid of rhizobia, are implied in the synthesis of lipochitooligosaccharides, also known as Nod factors (NF), which are responsible for launching the nodulation process^10^,^11^. Besides, other transcriptional regulators such as NolR, SyrM and NrcR also take part in the regulation of the *nod* genes^12^,^13^,^14^,^15^. In *Rhizobium leguminosarum* bv. *viciae*, flavonoid perception triggers transient increases in the concentration of intracellular calcium (Ca^2+^), which is essential for subsequent induction of the *nod* genes^16^. Interestingly, these Ca^2+^ transient peaks also occur in response to nitrogen starvation and environmental stresses in other prokaryotes^17^.

*Rhizobium tropici* CIAT 899 (hereafter CIAT 899) is the most successful symbiont of *Phaseolus vulgaris* (common bean) in tropical acid soils; it is also very promiscuous and capable of nodulating with a variety of legumes, with an emphasis on *Leucaena* spp.^18^,^19^. Genome sequencing of CIAT 899 allowed the identification of five different *nodD* genes and three different *nodA* genes in the symbiotic plasmid. The *nodD1* gene codes for the main activator of NF synthesis and is located adjacent to the *nodA1BC* operon^20^. Mutation of each *nodD* gene revealed that only *nodD1* is essential to nodulate *Leucaena leucocephala, Lotus japonicus* and *Macroptilium atropurpureum*. However, symbiosis with *P. vulgaris* and *L. burtii* is not blocked when the *nodD1* gene is mutated, indicating the existence of a complex symbiotic regulatory circuit in this bacterium^21^,^22^. Otherwise, the mutation in the *nodD2* gene provoked a significant decrease in nodule number in *P. vulgaris*, suggesting the putative implication of NodD2 in the symbiotic process^21^,^22^.

Main features of this broad host-range rhizobial strain include its high tolerance to environmental stresses such as high temperature, acidity and salinity^18^,^19^,^23^, and its capacity to produce a large variety of NF^24^. Surprisingly, CIAT 899 is able to produce these symbiotic molecules even in the absence of flavonoids under abiotic stress conditions such as acidity or high concentration of salt^25^,^26^,^27^. Interestingly, a transcriptomic analysis of CIAT 899 grown in the presence of apigenin (a CIAT 899 inducer flavonoid) or salt showed similarities in gene activation patterns not only for the *nod* genes, but also for other important symbiotic genes located downstream of their respective NB: *nodA1BCSUIJH* (NB1), *nodA2hsnTnodFE* (NB2), *nodM* (NB3), *y4wEF* (NB4) and two genes with still unknown functions (NB5). For this reason, the salt-dependent production of these symbiotic molecules could suggest a strategy of CIAT 899 to ensure nodulation under salt stress conditions^28^. Curiously, NF synthesis under salt stress is not regulated by NodD1^27^. However, the protein or proteins responsible for the activation of the *nod* genes in the presence of saline stress are currently unknown.

In this paper, we have unequivocally identified for the first time that a NodD protein (NodD2) is implied in the transcriptional activation of the nodulation genes under salt stress conditions in a flavonoid-independent manner. Besides, we have also demonstrated that both NodD1 and NodD2 control the entire NF synthesis in CIAT 899 and are necessary for a successful symbiosis with *P. vulgaris* and *L. burtii*.

## Results

### Mutation on the *nodD2* gene abolishes *nod* gene induction and the biosynthesis of NF under salt stress

As previously mentioned, Guasch-Vidal *et al*.^27^ demonstrated that even in the absence of inducer flavonoids, CIAT 899 was able to induce the synthesis of NF under salt stress conditions in a *nodD1*-independent manner. However, this bacterium harbours other four copies of the *nodD* gene in the symbiotic plasmid^20^ that could be responsible for *nod* genes activation in the presence of salt.

**Figure 1:**
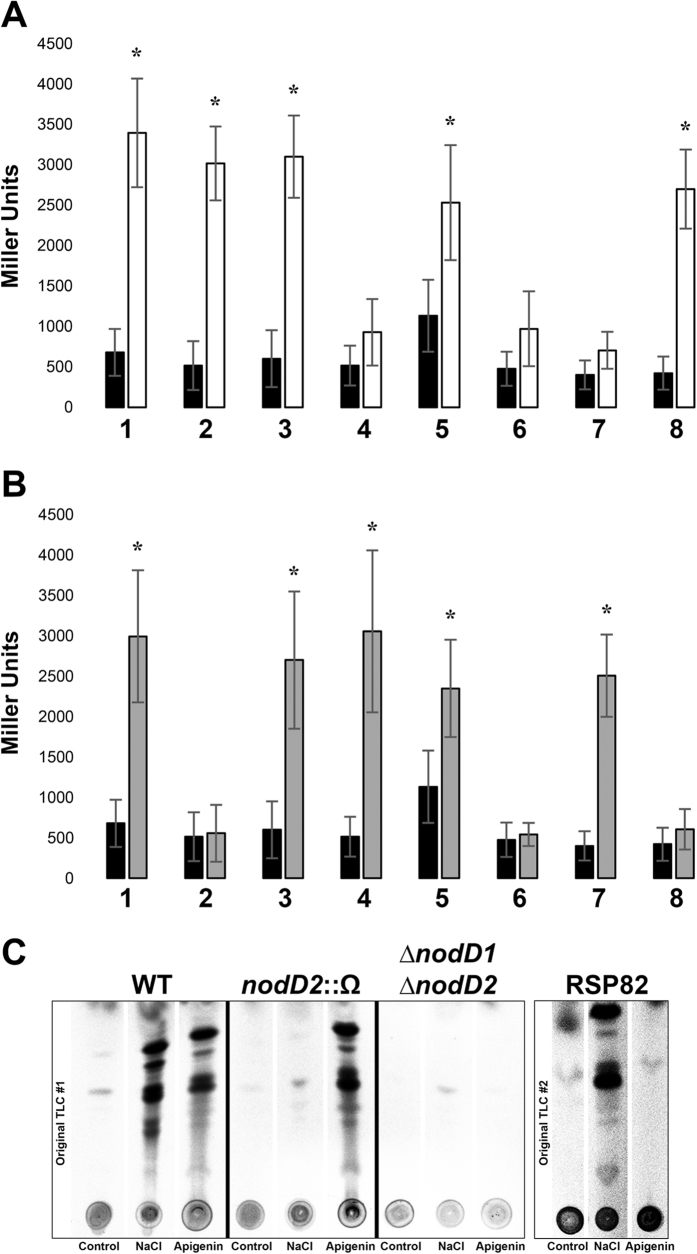
β-galactosidase activity of *R. tropici* CIAT 899 (1), RSP82 (*nodD1* mutant) (2), RSP82 (pMUS1398) (3), *nodD2*::Ω (4), *nodD2*::Ω (pMUS1396) (5), ∆*nodD1/*∆*nodD2* (6), ∆*nodD1/*∆*nodD2* (pMUS1397) (7) and ∆*nodD1/*∆*nodD2* (pMUS1395) (8) strains carrying plasmid pMP240 which contains the *R. leguminosarum* bv. *viciae nodA* promoter fused to the *lacZ* gene. Assayed conditions were YM medium (Control, black bars) YM supplemented with 300 mM NaCl (NaCl, white bars, (**A**) and YM supplemented with 3.7 μM apigenin (Apigenin, gray bars, (**B**). Strain parameters were individually compared with CIAT 899 grown in YM medium by using the Mann-Whitney non-parametric test. Values tagged by asterisks (*) are significantly different at the level of α = 5% **(C**). Thin-layer chromatography analysis of Nod factors produced by *R. tropici* CIAT 899 (1), RSP82 (2), *nodD2*::Ω (4) and ∆*nodD1/*∆*nodD2* (6) strains in the presence of ^14^C-labeled N-acetyl glucosamine.

To determine which of these five *nodD* genes induces the synthesis of NF under salt stress condition, plasmid pMP240 harbouring the *nodA* promoter of *R. leguminosarum* bv. *viciae* fused to the *lacZ* gene was transferred by conjugation to the CIAT 899 wild-type strain and to the *nodD* mutant derivative strains (Table S1). β-galactosidase assays showed similar activation levels in the presence of NaCl (300 mM) in all strains (Figure S2), with the exception of the strain affected in *nodD2 (nodD2*::Ω), in which the up-regulation of the *nodA* gene was only detected in cultures supplemented with apigenin but not in the presence of salt (Fig. 1A). To confirm these results, the *nodD2* gene was also mutated by in-frame deletion (∆*nodD2*) and the expression of the *nodA* gene was neither detected in this strain carrying plasmid pMP240 (data not shown). With respect to the *nodD1* mutant strain (RSP82) *nodA* activation was only detected in the presence of salt (Fig. 1A). As described in the materials and methods section, both mutant strains (RSP82 and *nodD2*::Ω) were complemented *in trans* and the complementation restored the wild-type β-galactosidase levels in apigenin and salt conditions, respectively (Fig. 1). These results suggest that NodD2 could be activating NF synthesis in the presence of salt in a pathway independent to those mediated by apigenin and NodD1. For this reason, β-galactosidase activity was also measured in the presence of both inducing molecules in a double ∆*nodD1/∆nodD2* mutant carrying plasmid pMP240. As expected, no induction of the *nod* genes in any assayed condition was detected. The double mutant was also complemented separately with the *nodD1* or the *nodD2* wild-type genes restoring the expression of *nodA* in the presence of apigenin or salt, respectively, confirming the results commented above (Fig. 1). There was still a residual *nodA* induction in the RSP82 strain in the presence of apigenin, the *nodD2*::Ω mutant in the presence of salt, and in the ∆*nodD1*/∆*nodD2* mutant in the presence of salt, but they were not statistically significant (Fig. 1).

The correlation between up-regulation of *nodA* expression and synthesis of NF was also demonstrated by reversed-phase thin layer chromatography (RP-TLC). The NF profiles of the wild-type, *nodD2 (nodD2*::Ω) and ∆*nodD1*/∆*nodD2* mutant strains were obtained by marking with ^14^C and further purification of these symbiotic molecules. Results obtained showed that NF production was abolished in the presence of high concentrations of salt when NodD2 was absent, while the double ∆*nodD1*/∆*nodD2* mutant did not produce NF in the presence of apigenin and under saline conditions (Fig. 1C). Therefore, these results confirm that NodD2 mediates the synthesis of NF in CIAT 899 under salt stress.

### RNA-seq analysis indicates that symbiotic genes are transcriptionally activated by NodD1 in the presence of apigenin and by NodD2 under salt stress

**Figure 2:**
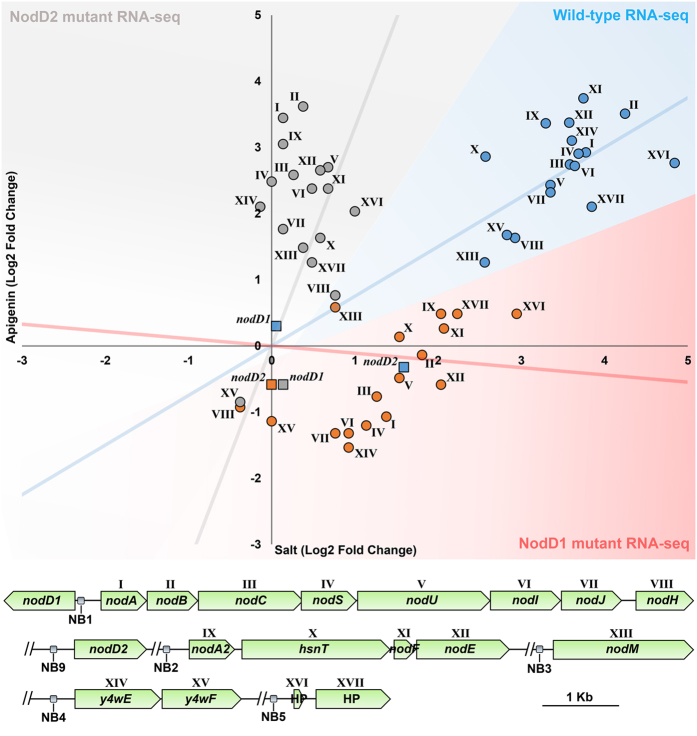
Scatterplot of the log_2_-transformed fold-change values of the *R. tropici* CIAT 899 *nod* genes and symbiotic-related genes obtained in the RNA-seq assay. Vertical and horizontal axis represent the values obtained in apigenin and salt conditions respectively. Nodulation genes were numbered with roman numerals (I to XVII) and represented in the Scatterplot. Blue circles represent the log_2_-transformed fold-change in our previous RNA-seq study of *R. tropici* CIAT 899 (Pérez-Montaño *et al*.^28^). Red circles represent the log_2_-transformed fold-change levels observed in RNA-seq of the *nodD1* mutant. Gray circles represent the log_2_-transformed fold-change values observed in the RNA-seq of the *nodD2* mutant. Values of the *nodD1* and *nodD2* genes are also present as squares and tagged with the own name of the gene. Trend lines of each RNA-seq assay are represented with its corresponding color. Black bar length = 1 Kb.

Plasmid pMP240 is a useful tool to measure the transcriptional activation of the *nodA* gene implied in NF synthesis. However, in our previous transcriptomic study of CIAT 899, at least 17 genes located in the symbiotic plasmid and controlled by 5 different NB are over-expressed in the presence of both inducer molecules apigenin and salt (Fig. 2)^28^. For this reason, to determine whether transcriptional activation of all these *nod* genes and the symbiotic-related genes under salt stress conditions is mediated by NodD2, 12 RNA-seq libraries were generated from wild-type, *nodD1* (RSP82 strain) and *nodD2 (nodD2*::Ω) mutant strains grown in the presence or absence of apigenin (3.7 μM) and salt (NaCl 300 mM) (two independent biological experiments for each condition). Quality control of each run, sample normalizations and statistical procedures were performed as previously described^28^ (Supplementary File 3). Differentially expressed genes for each strain and condition were obtained by comparing with gene expression levels of the wild-type strain grown under control conditions (Supplementary File 4). Data set was validated by *q*RT-PCR, obtaining in all cases positive correlation degrees in fold-change values between the *q*RT-PCR and the RNA-seq data (Supplementary File 5).

Focusing attention on these 17 genes, RNA-seq experiments indicated that their transcriptional activation was only detected in cultures supplemented with salt in the *nodD1* mutant background or apigenin in the case of the *nodD2* mutant background (Fig. 2), confirming the role of NodD2 in the transcriptional activation of these symbiotic genes under salt stress. Analysis of the induction values of each set of genes controlled by NB pointed to another interesting finding, the fold-change values of all symbiotic genes for the *nodD1* mutant in the presence of salt were lower than those observed for the wild-type strain in the same condition (Table 1). Indeed, two groups of symbiotic genes, *nodM* (NB3) and *y4wEF* (NB4) did not reach the threshold of induction values stablished for this study (±3 fold-change). This could be explained due to the fact that in the wild-type strain the expression of *nodD2* (NB9) was slightly up-regulated in the presence of salt but not in the presence of apigenin, which may suggest an initial role of NodD1 in the promotion of the transcriptional activation of *nodD2* under salt stress. Finally, the expression of *nrcR* transcriptional regulator, which activates the *nod* genes in presence of salt conditions in *R. tropici* CIAT 899, was reduced 2.3 times in the *nodD2*::Ω salt treatment respecting to the wild-type control condition, indicating that *nodD2* may activate also the *nrcR* gene.

In summary, transcriptomic analysis revealed that NodD2 mediates the transcriptional activation of all *R. tropici* CIAT 899 symbiotic genes under salt stress in a process which could be enhanced by NodD1.

### NodD1 and NodD2 control the entire production of the *R. tropici* CIAT 899 NF implied in symbiosis with legumes

Once unequivocally determined the key role of NodD1 and NodD2 in the biosynthesis of NF in the presence of both inducer molecules, the confirmation of the biological activity of these NF was evaluated. CIAT 899 produces low concentrations of NF even in the absence of apigenin or salt, being this biosynthesis strongly induced in the presence of both molecules^27^. Thus, it is not surprising that a single mutation of any *nodD* gene does not completely abolish NF production^21^,^22^. For this reason, the NF profile was firstly determined for the ∆*nodD1*/∆*nodD2* mutant by mass spectrometry. In this mutant background the production of NF was completely abolished in all conditions assayed (data not shown), indicating that NodD1 and NodD2 are crucial for the biosynthesis of NF in CIAT 899. Then, this result was confirmed by biological activity assays, in which the NF purified from cultures of CIAT 899, RSP82, *nodD2*::Ω and the ∆*nodD1*/∆*nodD2* mutant strains, induced or not with apigenin or salt, were added to common bean roots as previously described^27^ (Table 2). As expected, in the wild-type strain, nodule primordia were detected in common bean roots after 10 days post treatment, independently of the bacterial culture conditions being these primordia more abundant in those roots where the NF added were cultured in presence of any inducer. However, in the case of the double mutant strain, these symbiotic structures were not developed (Figure S6) (Table 2), presenting the common bean roots a phenotype similar to untreated plants. NF purified from the *nodD2*::Ω strain cultured in salt conditions also induced the formation of nodule primordia in *P. vulgaris* roots. However, the number of primordia was reduced 3-fold respecting the wild-type salt condition, whereas the nodule primordia observed in apigenin were similar between the wild-type and the *nodD2*::Ω strain. In turn, the number of primordia in the RSP82 strain was reduced in comparison to the wild-type, mainly in those NF purified from apigenin cultures.

Finally, once demonstrated that NodD1 and NodD2 control the entire NF production in CIAT 899, nodulation performance of the double ∆*nodD1*/∆*nodD2* mutant was also evaluated in *P. vulgaris* and *L. burtii*, two plants in which the absence of NodD1 reduced the nodule number but did not abolish the symbiotic process. Results showed that after 30 and 45 days of growth, respectively, nodules were absent in those plants inoculated with the double mutant strain, in concordance with results described above (Table 3). In order to confirm these results, the double mutant strain individually complemented with the wild-type *nodD1* and *nodD2* genes was also tested in nodulation assays. The presence of nodules in *P. vulgaris* and *L. burtii* roots was detected in both cases (Table 3). Thus, the nodulation machinery and therefore the synthesis of NF in the symbiosis with legume plants fully depended on both NodD1 and NodD2 transcriptional regulators in *R. tropici* CIAT 899.

## Discussion

Flavonoids released by legume roots are crucial for the symbiotic process. These molecules are recognized by rhizobia through binding to the transcriptional regulator NodD, which triggers the expression of the *nod* genes, implied in the biosynthesis of the bacterial Nodulation Factors (NF)^5^,^6^,^7^,^8^,^9^,^10^,^11^. This is an especial feature of rhizobia, since flavonoids have also been described as antimicrobial compounds for many bacteria, including plant pathogens^1^,^2^. Thus, rhizobia seem to redirect the stress caused by these phenylpropanoid compounds towards a beneficial relationship that culminates-in the formation of the nodule.

One of the most important symbionts of *P. vulgaris* is *R. tropici* CIAT 899^18^,^19^. This strain tolerates several environmental stresses and is characterized by its ability to produce a large variety of NF^24^, which are synthetized even in the absence of inducer flavonoids and in a NodD1-independent manner if bacteria are grown under salt stress conditions^27^. Recent transcriptomic data indicate that this bacterium shows similarities in its transcriptional activation patterns in the presence of both inducers not only for the *nod* genes, but also for other important symbiotic genes, suggesting a strategy to overcome salt stress conditions towards symbiosis^28^. In our study we have determined that NodD2 is the transcriptional regulator responsible of the up-regulation of these symbiotic genes under salt stress.

First, we demonstrated that NodD2 is responsible for the transcriptional activation of the *nodA* gene in the presence of high concentrations of salt. This result correlates with the abolishment of the NF production under this inducing condition (Fig. 1). This finding is not enough to ensure the role of NodD2 in the activation of the CIAT 899 symbiotic genes, since at least 17 genes, controlled by 5 different NBs, are up-regulated in the presence of both inducing molecules^28^. Although not all genes are fully characterized, it is well known that many of them are involved in NF production (*nodA1BCSUIJH, nodA2hsnTnodFE* and *nodM* genes) or in the synthesis of the phytohormone indole-3-acetic acid (*y4wEF* genes). For this reason, the RNA-seq study was decisive to ascertain the main role of NodD2 in the promotion of symbiosis under salt stress conditions (Fig. 2) (Table 1) (Supplementary Files 3 4 5). Thus, two initially unfavorable conditions are activating the same symbiotic machinery in CIAT 899, maybe in a strategy to overcome both bacterial stresses by means of the establishment of a symbiotic association. Besides, it is possible that this strategy is not exclusive of salt stress conditions, since other environmental stresses such as acidity or osmotic pressure (high concentration of mannitol) also induce the *nodA1BC* operon in CIAT 899 (our unpublished data). Interestingly, other bacterial stressing compounds such as trigonelline and stachydrine, two alkaloids released by alfalfa seeds, also activate the NodD2 protein of *Sinorhizobium meliloti*^29^. Further experiments will be required to elucidate whether several events in the rhizosphere, such as a high osmotic pressure, are inducing the symbiotic genes at the same level of a plant-released flavonoid.

**Figure 3:**
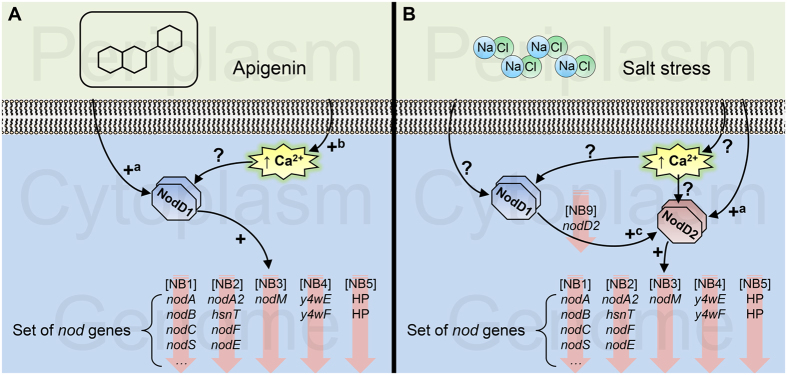
Proposed model to explain the regulation of the expression of the *nod* genes under apigenin (**A**) and salt stress (**B**) conditions in *Rhizobium tropici* CIAT 899. In the presence of high concentration of salt, NodD1 binds to NB9 increasing the expression of *nodD2*. (+) Indicates confirmed up-regulation, (+^a^) indicates that flavonoids or salt activate NodD1 and NodD2, respectively, whereas other factors may also be involved in the activation, (+^b^) indicates up-regulation confirmed in *Rhizobium leguminosarum* bv. *viciae*, (+^c^) indicates putative up-regulation according to our transcriptomic CIAT 899 data, where in the presence of high concentration of salt, NodD1 binds to NB9 increasing the expression of *nodD2* expression. Finally, (?) indicates hypothetic up-regulation.

Finally, transcriptomic analyses indicated that despite the fact that NodD1 is not involved in the up-regulation of symbiotic genes under salt stress, there is one gene up-regulated via NodD1 and salt and located downstream of a NB, the *nodD2* gene. This finding indicates that NodD1 could be increasing *nodD2* expression to enhance the concentration of NodD2 and consequently the production of NF in the presence of high concentrations of salt (Fig. 3). However, how salt is activating NodD2 is uncertain. It has been reported that in *R. leguminosarum* transient increases of cytoplasmatic Ca^2+^ are generated in the presence of flavonoids^16^. Thus, it would be possible that environmental stresses could also be generating a transient intracellular Ca^2+^ increase or activating other unknown regulatory elements, that would activate the symbiotic machinery generating also certain activation of NodD1, which would bind to the NB located upstream of the *nodD2* gene, increasing the concentration of NodD2.

The link between NodD1 and NodD2 was also confirmed in assays of biological activity and nodulation tests, since both transcriptional regulators are crucial for the production of symbiotic NF in CIAT 899, which is directly related with the ability of this bacterium to nodulate with *P. vulgaris* and *L. burtii* (Tables 2 and 3). Here we reported the *in vivo* role of NodD2 since it is necessary the absence of both NodD1 and NodD2 to abolish the nodulation processes in these legumes. These results indicated that NodD2 had a biological role in the induction of the nodulation genes independently of the classic activation of these genes in the presence of the corresponding flavonoids. However, the concentration of salt employed in this work (300 mM) in the *in vitro* experiments cannot be used in the nodulation assays because legumes used in this work cannot grow in salt concentrations higher than 25 mM (our unpublished data). Thus, further experiments will determine which biological condition/s in the rhizosphere is inducing the activation of the *nod* genes through NodD2.

In conclusion, this is the first time in which it is demonstrated that a NodD protein is directly implied in the transcriptional activation of all symbiotic genes and in the synthesis of NF under an abiotic stress condition. Absence of NodD2 but not of NodD1 results in an absence of NF under saline conditions. However, we have observed in the RNAseq analyses that the nodulation genes induced in saline conditions in the wild-type strain CIAT 899 were induced to a higher rate than the corresponding genes induced in the *nodD1* mutant. These results could indicate that NodD1 could somehow be involved in the activation of the NF synthesis under saline conditions. In these conditions, NodD2 activates the transcription of the *nod* genes, and we cannot discard that NodD2 also activate the *nodD1* gene, and NodD1 in turn, activate the expression of the *nod* genes. Thus, the mechanism by which salt is directly or indirectly promoting the activation of NodD2 remains unclear. Future efforts should be focused in the molecular understanding of this special response of CIAT 899 under salt stress conditions.

## Material and methods

### Bacteria growth conditions, plasmids, mutant obtaining

All strains and plasmids used in this work are listed in Table S1. *R. tropici* CIAT 899 strains were grown at 28 °C on tryptone yeast (TY) medium^30^, B- minimal medium^31^ or yeast extract mannitol (YM) medium^32^, supplemented when necessary with apigenin 3.7 μM or with NaCl 300 mM. *Escherichia coli* strains were cultured on LB medium^33^ at 37 °C. When required, the media were supplemented with the appropriate antibiotics as previously described^34^. The ∆*nodD2* and the double ∆*nodD1/*∆*nodD2* deletion mutants were made by overlapping PCR extension as previously described^35^, where 819 pb and 885 pb length were deleted from *nodD1* and *nodD2*, respectively. The deleted DNA fragments were firstly cloned in the pK18mob*sacB* vector obtaining plasmids pMUS1397 and pMUS1395, respectively. Then, plasmids were transferred by conjugation and integrated in the genome of CIAT 899 by single recombination. Finally, a homologue recombination event in which the wild-type copy of the gene together with plasmid pK18mob*sacB* were lost, was selected. Mutated strains were confirmed by PCR and DNA-DNA hybridization. Moreover, both *nodD2* mutant strains were complemented: plasmid pBBR1-MCS-5 containing the wild-type *nodD2* gene (pMUS1396) was transferred to the *nodD2*::Ω mutant strain generating the *in trans* complemented strain *nodD2*::Ω (pMUS1396). The ∆*nodD2* mutant was complemented *in cis* by integration of plasmid pK18mob*sacB* containing the *nodD2* wild-type copy (pMUS1395) in the genome, obtaining the ∆*nodD2* (pMUS1395) strain. Besides, the *nodD1* mutant (RSP82 strain) was also complemented *in trans* with plasmid pBBR1-MCS-5 containing the wild-type *nodD1* gene (pMUS1398). Finally, the double ∆*nodD1/*∆*nodD2* deletion mutant was also complemented separately with a copy of the *nodD1* and *nodD2* wild-type gene. For that, plasmid pMUS1395 and the *nodD1* gene cloned into plasmid pK18mob*sacB* (pMUS1397) were integrated in the genome of the double mutant strain by conjugation and simple recombination, respectively, obtaining the ∆*nodD1*/∆*nodD2* (pMUS1397) and the ∆*nodD1*/∆*nodD2* (pMUS1395) strains. Primers employed in this study are listed in Table S7.

The parental and derivative strains were deposited at the culture collection of the Department of Biology of the Universidad de Sevilla and at the Diazotrophic and Plant Growth Promoting Bacteria Culture Collection of Embrapa Soja (WFCC Collection #1213, WDCM Collection #1054).

### Determination of β-galactosidase activity

To determinate the β-galactosidase activity, wild-type, mutant and complemented strains were conjugated with the plasmid pMP240 which contains the transcriptional fusion between the *R. leguminosarum* bv. viciae *nodA* promoter and the *lacZ* gene (Table S1)^36^. Assays of β-galactosidase activity from CIAT 899 and derivative strains were carried out as described by Zaat *et al*.^37^. Units of β-galactosidase activity were calculated according to Miller^38^. The experiments were repeated three times, with six replicates each time.

### RP-TLC analysis of NF

Reversed-phase thin layer chromatography analyses were performed according to Spaink *et al*.^31^. Briefly, *R. tropici* CIAT 899 was grown on B^-^ minimal medium, supplemented when necessary with inducing molecules. For the radiolabeling of Nod factors, 0.2 μCi of N-acetil-D-[1-^14^C]-glucosamine (specific activity 0.05 mCi) (PerkinElmer) was used. Cultures of 1 mL were grown to the end of the exponential growth phase and the supernatant was extracted with water-saturated butanol. The butanol fraction was evaporated to dryness and the resulting powder dissolved in 40 μl of water-saturated butanol. This solution (10 μL) was applied to the TLC plate (RP-18F254S) (Merck), where the Nod factors were separated with 50% acetonitrile/H_2_O (vol/vol) as the mobile phase. TLC plates were exposed to a Fuji BAS-IIIs film for 10 days and the image was digitalized using the Phosphor-image system (Fuji).

### Identification of NF and biological activity assays

NF purification and LC-MS/MS analyses were performed as previously described^21^,^22^ by growing the wild-type and the derivative mutant strains in B^−^ minimal medium, supplemented when required with NaCl 300 mM or apigenin 3.7 μM.

The purified NF were used for biological activity assays. Thus, common bean seeds were surface-sterilized and mounted in test tubes on a curled wire with the roots in 25 mL of Farhaeus medium^39^. Roots were shielded from light, and plants were grown for 10 days. Chamber conditions were 16 h at 26 °C in the light and 8 h and 18 °C in the dark, with 70% of humidity. To determine the presence of pseudonodules, roots were cleared with sodium hypochlorite and stained with methylene blue using the method of Truchet *et al*.^40^. Each experiment was repeated three times with six plants for each treatment.

### Nodulation assays

For the evaluation of the symbiotic phenotypes, the wild-type, the mutants and the complemented strains were grown in YM medium until the concentration of 10^9^ cells mL^−1^. Surface-sterilized seeds of *P. vulgaris* and *L. burtii* were pre-germinated and placed on sterilized Leonard jars and test tubes respectively, containing Farhaeus N-free solution^39^. Germinated seeds were then inoculated with 1 mL of bacterial culture. Growth conditions were 16 h at 26 °C in the light and 8 h and 18 °C in the dark, with 70% of humidity. Nodulation parameters were evaluated after 30 days for *P. vulgaris* or 45 days for *L. burtii*. Shoots were dried at 70  °C for 48 h and weighed. Nodulation experiments were performed three times.

### RNA extraction

Total RNA was isolated using a High Pure RNA Isolation Kit (Roche), according to the manufacturer’s instructions. Verification of the amount and quality of total RNA samples was carried out using a Nanodrop 1000 spectrophotometer (Thermo Scientific) and a Qubit 2.0 Fluorometer (Invitrogen). Two independent total RNA extractions were obtained for each condition.

### Quantitative reverse transcription PCR

Results obtained in the RNA-seq analysis were validated by quantitative reverse transcription PCR (*q*RT-PCR) of 13 selected nodulation genes which represented differentially and non-differentially expressed genes in the presence of apigenin and salt. Total RNA was isolated using a High Pure RNA Isolation Kit (Roche) and RNAase Free DNA Set (Qiagen), according to the manufacturer’s instructions. This (DNA-free) RNA was reverse transcribed into cDNA using a QuantiTec Reverse Transcription Kit (Qiagen). Quantitative PCR was performed using a LightCycler 480 (Roche) with the following conditions: 95 °C, 10 min; 95 °C, 30 s; 50 °C, 30 s; 72 °C, 20 s; forty cycles, followed by the melting curve profile from 60 to 95 °C to verify the specificity of the reaction. The *R. tropici* CIAT 899 16S rRNA gene was used as an internal control to normalize gene expression. The fold-changes of two biological samples with three technical replicates of each condition were obtained using the ΔΔCt method^41^. Selected genes and primers are listed in S5.

### RNA sequencing

Ribosomal RNA was depleted using a MICROB Express Bacterial mRNA Purification kit (Ambion), following the manufacturer’s protocol. Integrity and quality of the ribosomal depleted RNA was checked with Agilent Bioanalyzer 2100 (Agilent Technologies). RNA sequencing was carried out by Sistemas Genómicos (https://www.sistemasgenomicos.com/web_sg/) with the Next Generation Sequence (NGS) platform Illumina using the Illumina HiSeq 2000 sequencing instrument (Illumina). Ribosomal-depleted samples were used to generate whole transcriptome libraries following the manufacturer’s recommendations for sequencing on this NGS platform. Amplified cDNA quality was analyzed by the Bioanalyzer 2100 DNA 1000 kit (Agilent Technologies) and quantified using the Qubit 2.0 Fluorometer (Invitrogen). The RNA-seq data discussed in this work have been deposited in the Sequence Read Archive of NCBI under the accession number PRJNA326592.

### Mapping of the RNA-seq data

The initial whole transcriptome paired-end reads obtained from sequencing were mapped against the latest version of the *R. tropici* CIAT 899 genome (http://www.ncbi.nlm.nih.gov/genome/?term=Rhizobium_tropici_CIAT_899) using the Life Technologies mapping algorithm version 1.3 (http://www.lifetechnologies.com/). Low-quality reads were eliminated using Picard Tools software version 1.83, remaining only high quality reads.

### Assessment of differentially expressed genes

Gene prediction was estimated using the cufflinks method^42^ and the expression levels were calculated using the htseq software, version 0.5.4p3^43^. This method eliminates multimapped reads, considering only unique reads for the gene expression estimation. The edge method version 3.2.4 was applied for differential expression analysis among conditions^44^. This method uses a Poisson model to estimate the variance of the RNA-seq data for differential expressions, and relies on different normalized processes based on depth global samples, CG composition and length of genes. Differentially expressed genes were established in those genes with a fold-change lower or higher than −3 or 3, respectively, with a *p* value adjust to 0.15^45^,^46^.

## Additional Information

**How to cite this article:** del Cerro, P. *et al*. The *Rhizobium tropici* CIAT 899 NodD2 protein regulates the production of Nod factors under salt stress in a flavonoid-independent manner. *Sci. Rep.* **7**, 46712; doi: 10.1038/srep46712 (2017).

**Publisher's note:** Springer Nature remains neutral with regard to jurisdictional claims in published maps and institutional affiliations.

## Supplementary Material

Supplementary files 1-7

Supplementary Dataset S4

## Figures and Tables

**Table 1:** Fold-change values of the *R. tropici* CIAT 899 genes located downstream *nod* boxes.

NB	Locus-tag (gene name)	Function	CIAT 899 salt^a,b^	CIAT 899 apigenin^a,b^	*nodD1* mutant salt^a^	*nodD1* mutant apigenin^a^	*nodD2* mutant salt^a^	*nodD2* mutant apigenin^a^
1	cds215 to 224/RTCIAT899_PB1300 to RTCIAT899_PB01340/*nodABCSUIJH*	NF production	**13.66 to 7.6**	**8.73 to 3.09**	3.46^*^ **to −1.31**	**−**2.08 to **−**1.94	**−**1.12 to **−**1.74	**10.85 to 1.73**
2	cds177 to 180/RTCIAT899_ PB01095 to RTCIAT899_PB01110/*nodA2hsnTnodFE*	NF production	**9.81 to 11.94**	**10.3 to 10.37**	**4.13 to 4.10**	1.40 to **−**1.51	1.07 to 1.46	**8.29 to 6.34**
3	cds450/RTCIAT899_ PB02710/*nodM*	NF production	**5.85**	2.43	1.67	**−**1.51	1.31	2.79
4	cds95 to 94/RTCIAT899_PB00575 to RTCIAT899_PB00565/*y4wEF*	Synthesis of IAA	**12.17 to 7.11**	**8.57 to 3.2**	1.87 to 1.04	**−**2.94 to **−**2.22	**−**1.12 to **−**1.3	**4.25 to −1.83**
5	cds263 to 262/RTCIAT899_PB01550 to RTCIAT899_PB01545/HP	Unknown	**28.65 to 14.37**	**6.75 to 4.26**	**7.72 to 4.73**	1.36 to 1.42	1.96 to 1.42	**4.1 to 2.35**
9	cds173/RTCIAT899_PB01570/*nodD2*	Transcriptional regulation	**3.01**	**−**1.25	1.02	**−**1,52	**—**	—

Transcriptional activation (3-fold induction with respect to CIAT 899 non-induced cultures) of several *nod* box controlled operons was demonstrated by RNA-seq experiments in the presence of both inducer molecules (fold-change values in bold). HP: gene that codes for a hypothetical protein.

^a^Fold induction with respect to CIAT 899 non-induced cultures. Values correspond to fold-change values of the first and last gene of the cluster.

^b^Data obtained from Pérez-Montaño *et al*.^28^.

^*^3.46 correspond to fold induction of the *nodB* gene.

**Table 2:** Number of nodule primordia in response to inoculation of common bean with the purified Nod factors of *R. tropici* CIAT 899 and derivative strains.

Strains	Control	NaCl (300 mM)	Apigenin (3.7 μM)
*R. tropici* CIAT 899	12.5 ± 5.69	23.14 ± 3.48*	35.29 ± 12.88*
RSP82	4.73 ± 3.85*	13.86 ± 5.7	5.3 ± 4.05*
*nodD2*::Ω	8.33 ± 4.72	8.22 ± 4.46	41.17 ± 5.03*
∆*nodD1/*∆*nodD2*	0 ± 0*	0 ± 0*	0 ± 0*
none	0 ± 0*	0 ± 0*	0 ± 0*

Data represent means ± S.D. of six plant jars. Each jar contained one plant. *P. vulgaris* plants were evaluated 10 days after inoculation.

The CIAT 899 derivatives and no inoculated parameters were individually compared with the parental strain by using the Mann-Whitney non-parametric test. Values tagged by asterisk (*) are significantly different (α = 5%).

**Table 3:** Plant responses to inoculation of common bean and *Lotus burtii* with *R. tropici* CIAT 899 and derivative strains.

Strains	*P. vulgaris*^a^		*L. burtii*^a^	
	Nodule number	Shoot dry weight (mg)	Nodule number	Shoot dry weight (mg)
*R. tropici* CIAT 899	225.67 ± 30.25	1890 ± 570	14.87 ± 5.79	46.7 ± 18.7
RSP82^b^	38.11 ± 11.14*	1420 ± 350	5.14 ± 2.13*	40 ± 20
*nodD2*::Ω^b^	95.08 ± 38.26*	1030 ± 270	12.1 ± 5.45	30 ± 10.1
∆*nodD1/*∆*nodD2*	0 ± 0*	560 ± 190*	0 ± 0*	8.7 ± 3.8*
∆*nodD1/*∆*nodD2* (pMUS1397)	109.41 ± 33.47*	1180 ± 310	10 ± 4.22	38 ± 19.2
∆*nodD1/*∆*nodD2* (pMUS1395)	56.5 ± 16.03*	1220 ± 390	8 ± 4.89	32 ± 14.9
none	0 ± 0*	500 ± 40*	0 ± 0*	4.8 ± 1.3*

^a^Data represent means ± S.D. of six plant jars. Each jar contained two plants. *P. vulgaris* plants were evaluated 30 days after inoculation. *Lotus burtii* plants were evaluated 45 days after inoculation.

^b^Data obtained from del Cerro *et al*.^22^.

The CIAT 899 derivatives and no inoculated parameters were individually compared with the parental strain by using the Mann-Whitney non-parametric test. Values tagged by asterisk (*) are significantly different (α = 5%).
