# Supplementary material for: The Rhizobium tropici CIAT 899 NodD2 protein regulates the production of Nod factors under salt stress in a flavonoid-independent manner
Source: Sci Rep. 2017 May 10;7:46712. doi: 10.1038/srep46712 (PMC5424341; doi:10.1038/srep46712)

Title: The *Rhizobium tropici* CIAT 899 NodD2 protein regulates the production of Nod factors under salt stress in a flavonoid-independent manner.

Authors: Pablo del Cerro[1], Francisco Pérez-Montaño[1], Antonio Gil-Serrano[3], Francisco Javier López-Baena[1], Manuel Megías[1], Mariangela Hungria[2], Francisco Javier Ollero[1*#].

[1]Departamento de Microbiología, Facultad de Biología, Universidad de Sevilla. Sevilla, Spain. [2]Embrapa Soja, Londrina, Paraná, Brazil. [3]Departamento de Química Orgánica, Facultad de Química, Universidad de Sevilla. Sevilla, Spain.

# Address correspondence to Francisco Javier Ollero, Calle Profesor García González, s/n, 41012 Sevilla. E-mail: fjom@us.es.

* Corresponding author

Running title: NodD2 activates *nod* genes under salt stress.

Table S1. Bacterial strains and plasmids employed in this study.

Strain or plasmid	Derivation and relevant properties	Source or reference
Rhizobium tropici CIAT 899	Wild-type strain (Rif^{R})	(**19**)
pMP240	Transcriptional fusion between the *R. leguminosarum* bv. *viciae nodA* promoter and the *lacZ* gene	(**36**)
pK18mob*sacB*	Rhizobial suicide vector containing the *sacB* counter-selection gene (Km^{R})	(**46**)
pBBR1-MCS-5	Expression vector (Gm^{R})	(**45**)
pMUS1395	*nodD2* gene cloned into the pK18mob*sacB* plasmid (Km^{R})	This work
pMUS1396	*nodD2* gene cloned into the pBBR1-MCS-5 plasmid (Gm^{R})	This work
pMUS1397	*nodD1* gene cloned into the pK18mob*sacB* plasmid (Km^{R})	This work
pMUS1398	*nodD1* gene cloned into the pBBR1-MCS-5 plasmid (Gm^{R})	This work
RSP82	*R. tropici* CIAT 899 carrying a Km-resistant (Km^{R}) cassette inserted into the *nodD1* gene	(**27**)
RSP82 (pMUS1398)	RSP82 strain complemented *in trans* by the pMUS1398 plasmid	This work
nodD2::Ω	*R. tropici* CIAT 899 carrying a Spc-resistant (Spc^{R}) cassette inserted into the *nodD2* gene	(**21**)

nodD2::Ω (pMUS1396)	*nodD2*::Ω mutant complemented *in trans* by the pMUS1396 plasmid	This work
Δ*nodD2*	*R. tropici* CIAT 899 *nodD2* deletion mutant	This work
Δ*nodD2* (pMUS1395)	Δ*nodD2* mutant complemented *in cis* by the pMUS1395 plasmid	This work
RSP82 (pMP240)	RSP82 strain carrying plasmid pMP240 (Tc^R)	This work
nodD2::Ω (pMP240)	*nodD2*::Ω mutant carrying plasmid pMP240 (Tc^R)	This work
nodD3::Ω (pMP240)	*nodD3* mutant harboring plasmid pMP240 (Tc^R)	This work
nodD4::Ω (pMP240)	*nodD4* mutant harboring plasmid pMP240 (Tc^R)	This work
nodD5::Ω (pMP240)	*nodD5* mutant harboring plasmid pMP240 (Tc^R)	This work
Δ*nodD2* (pMP240)	Δ*nodD2* mutant carrying plasmid pMP240 (Tc^R)	This work
Δ*nodD1*/Δ*nodD2*	*R. tropici* CIAT 899 double *nodD1*/*nodD2* deletion mutant	This work
Δ*nodD1*/Δ*nodD2* (pMP240)	Δ*nodD1*/Δ*nodD2* mutant carrying plasmid pMP240 (Tc^R)	This work
Δ*nodD1*/Δ*nodD2* (pMUS1397)	Δ*nodD1*/Δ*nodD2* mutant complemented *in cis* with the *nodD1* wild type gene present in the pMUS1397 plasmid (Km^R)	This work
Δ*nodD1*/Δ*nodD2* (pMUS1395)	Δ*nodD1*/Δ*nodD2* mutant complemented *in cis* with the *nodD2* wild type gene present in the pMUS1395 plasmid (Km^R)	This work

Δ*nodD1*/Δ*nodD2* (pMP240) (pMUS1397)	Δ*nodD1*/Δ*nodD2* mutant carrying plasmid pMP240 (Tc^R) and complemented *in cis* with the *nodD1* wild type gene present in the pMUS1397 plasmid (Km^R)	This work
Δ*nodD1*/Δ*nodD2* (pMP240) (pMUS1395)	Δ*nodD1*/Δ*nodD2* mutant carrying plasmid pMP240 (Tc^R) and complemented *in cis* with the *nodD2* wild type gene present in the pMUS1395 plasmid (Km^R)	This work

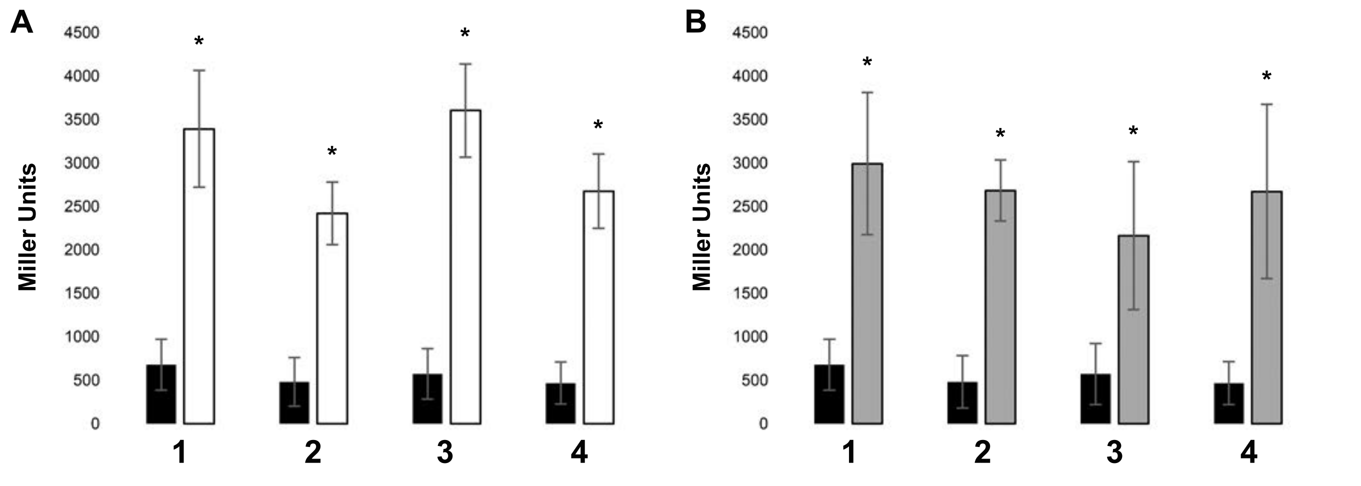
A
B
Miller Units
4500
4000
3500
3000
2500
2000
1500
1000
500
0
*
1
2
3
4

Supplementary file 2 legend. β-galactosidase activity of *R. tropici* CIAT 899 (**1**), *nodD3*::Ω (**2**), *nodD4*::Ω (**3**) and *nodD5*::Ω (**4**) strains carrying plasmid pMP240 which contains the *R. leguminosarum* bv. *viciae nodA* promoter fused to the *lacZ* gene. Assayed conditions were YM medium (Control, black bars), YM supplemented with 300 mM NaCl (NaCl, white bars, **A**) and YM supplemented with 3.7 µM of apigenin (Apigenin, gray bars, **B**). Strain parameters were individually compared with CIAT 899 grown in YM medium by using the Mann-Whitney non-parametric test. Values tagged by asterisks (*) are significantly different at the level of $\alpha = 5\%$.

Supplementary file 3. General features of the total sequenced and mapped reads.

Total Number of Reads: Reads were mapped using a Bayesian inference using Cufflinks v2.11 software. Worst quality reads were removed by means of Picard Tools.

Sample condition	#Mapped Reads
D1_API_1	48156485
D1_API_2	50347309
D1_SALT_1	42616476
D1_SALT_2	50481078
D1_1	56635661
D1_2	51403320
D2_API_1	41943049
D2_API_2	41362670
D2_SALT_1	52452032
D2_SALT_2	48337829
D2_1	48988155
D2_2	53167166

Two biological and independent experiments were carried out for each condition.

Quality Read Controls: Three different controls were performed to ensure the quality of reads: GC content, duplicate distribution and distribution respect genetic coordinates.

- *GC content*: distribution of GC content on mapped reads. A normal distribution around 45-55% is expected.

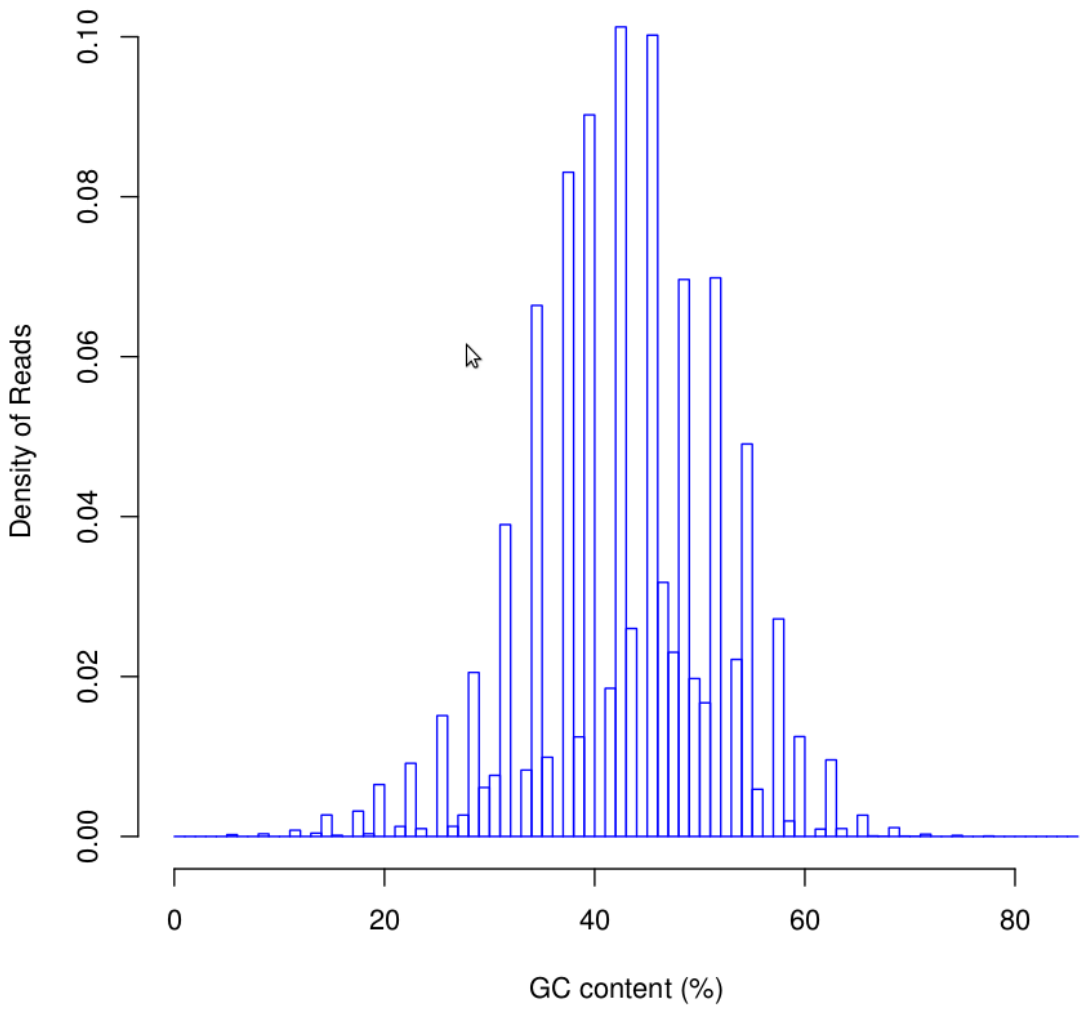

- *Duplicate distribution*: common distribution of duplicates in a RNA-Seq experiment shows a small number of reads with high levels of duplicates and a high number of reads with low levels of duplicates. All samples presented optimal values of duplicate distributions.

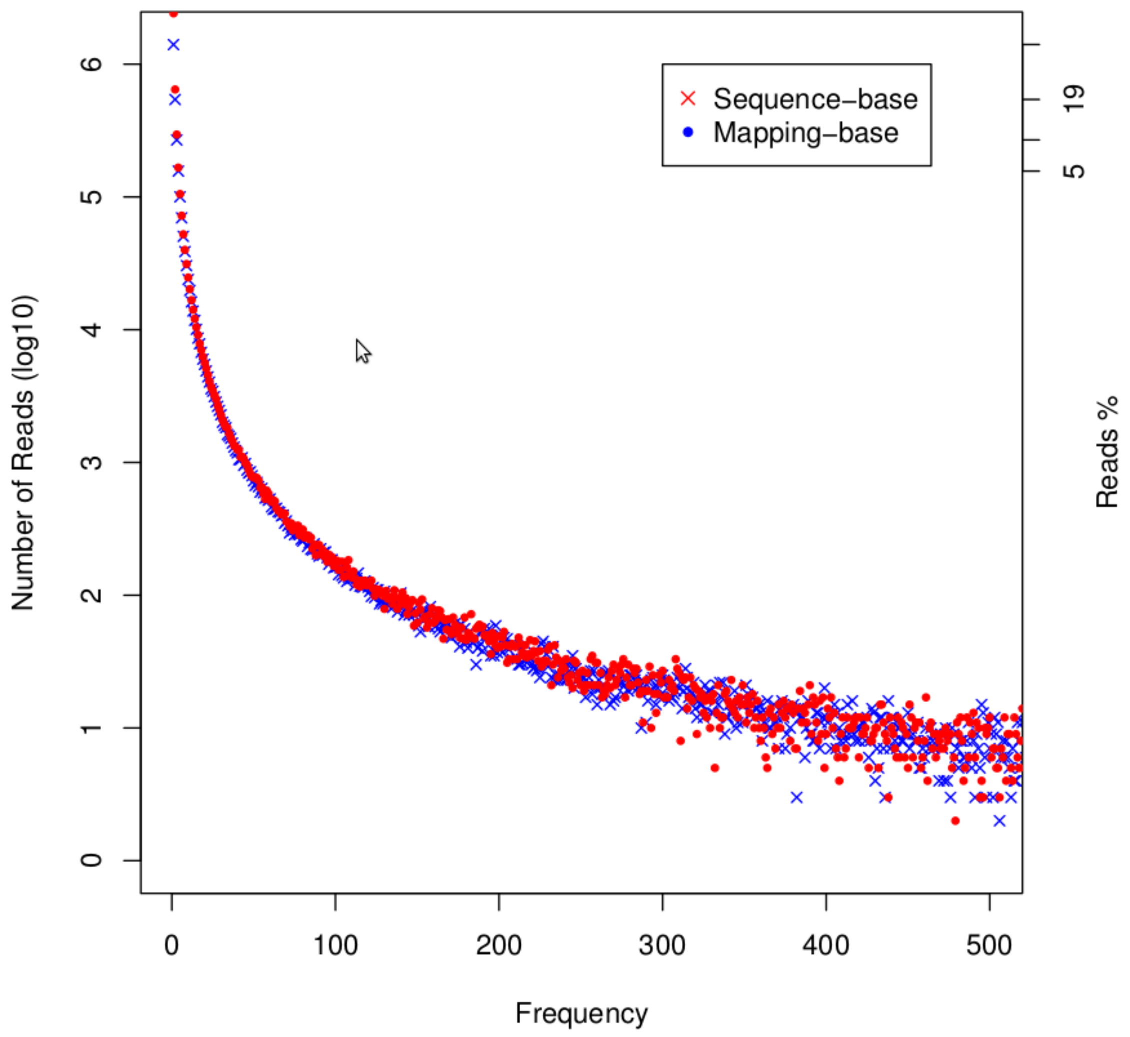

- *Distribution respect genetic coordinates*: centralization around 50 percentile of gene body is expected in high quality samples. Read concentration in both ends could be indicative of RNA degradation.

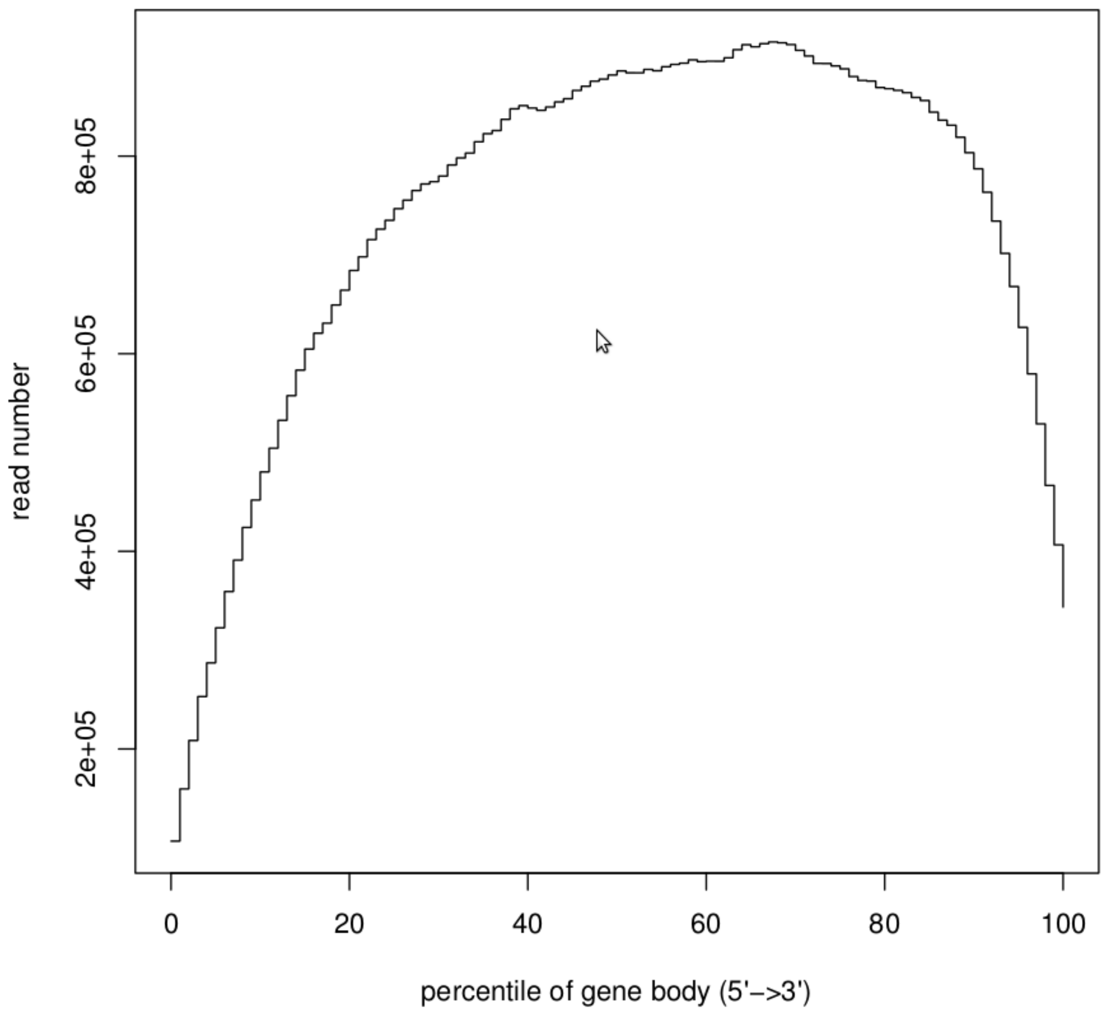

Normalization: normalization is needed to avoid statistical deviations due to differences in library sizes. Each mutant samples were compared with control samples obtained in Pérez-Montaño *et al.*, (2016). These RNA-seq data were deposited in the Sequence Read Archive of NCBI under the accession number SRP067561.

Number of reads per gene for control samples of *nodD1* mutant and wild-type strains **before** normalization.

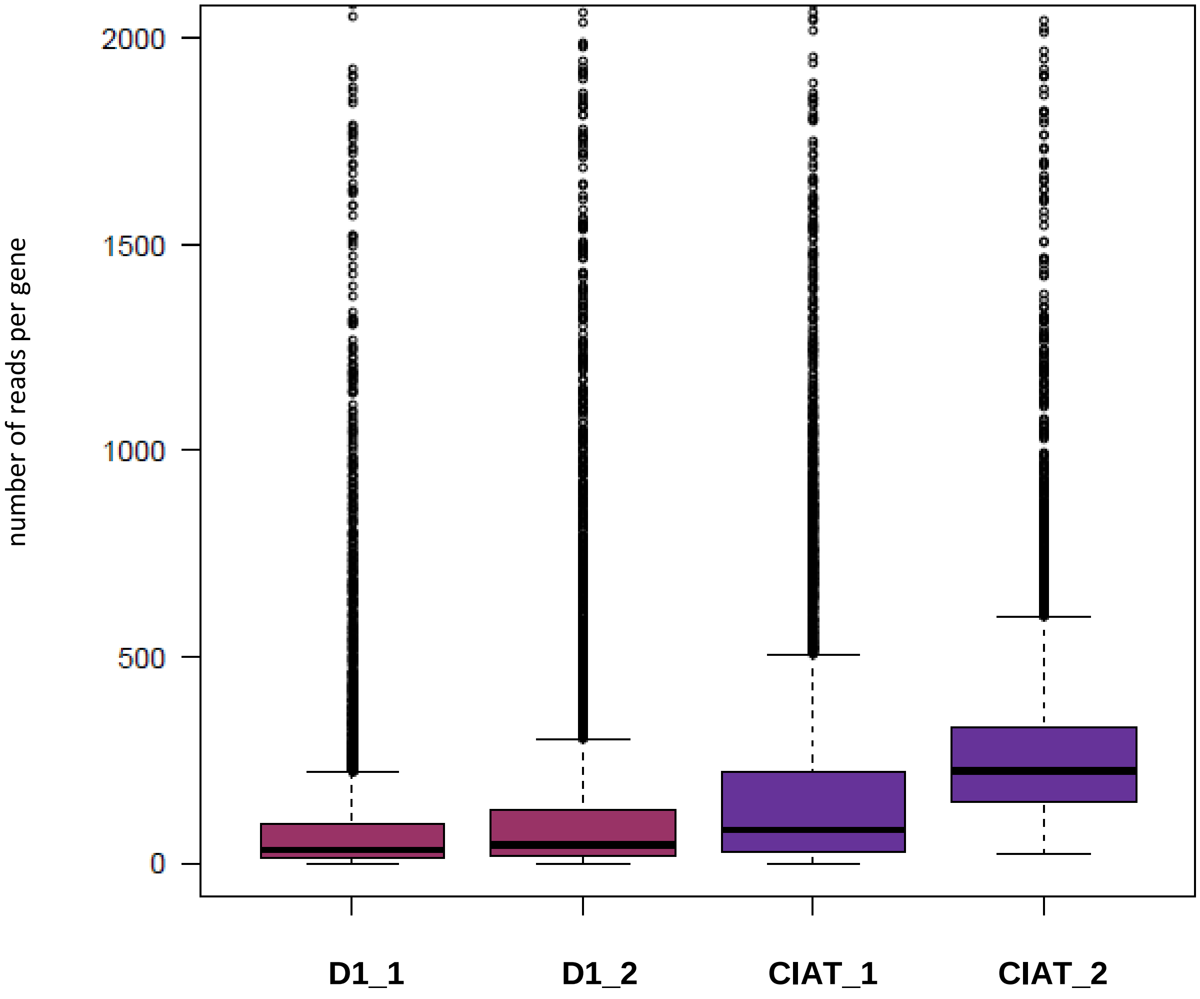

Number of reads per gene for control samples of *nodD1* mutant and wild-type strains **after** normalization.

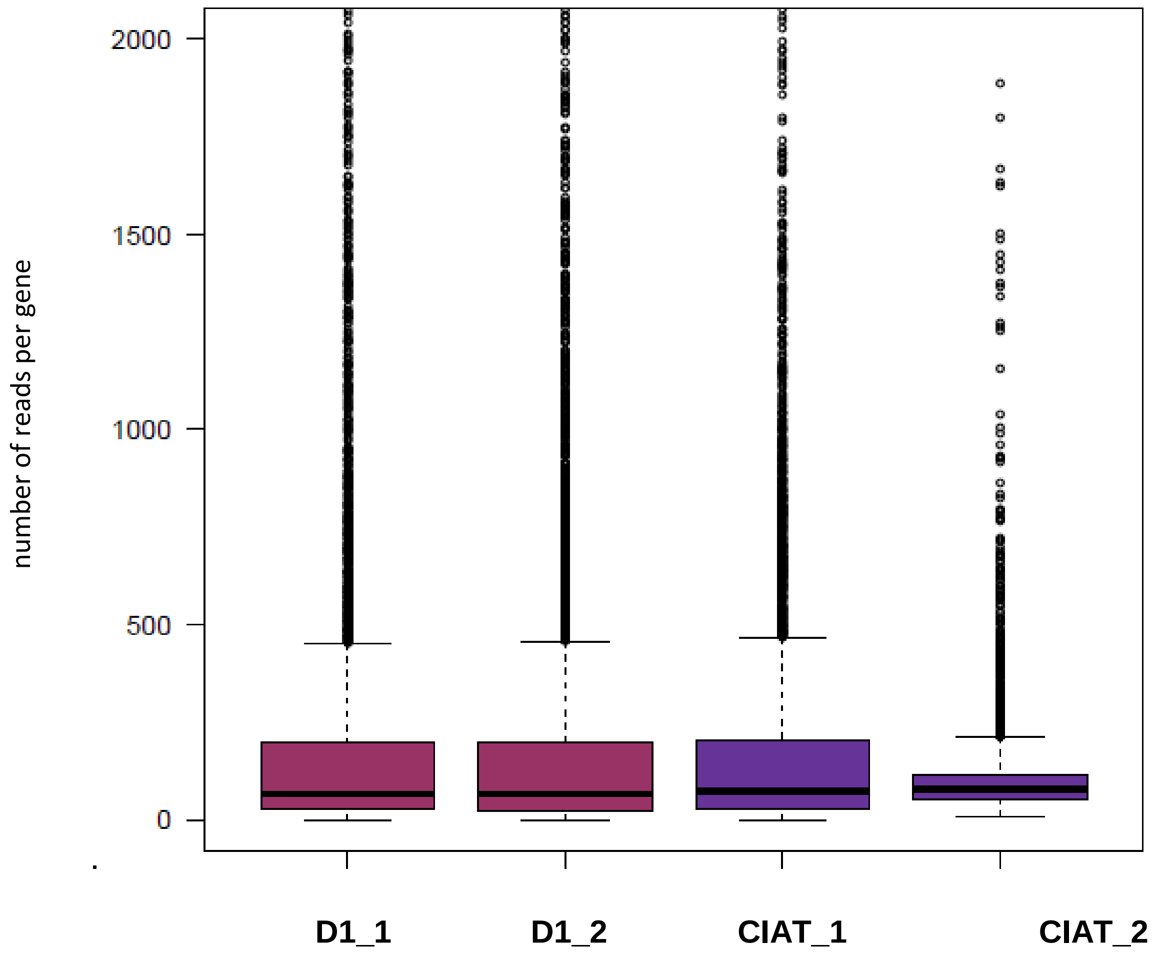

.

Number of reads per gene for salt samples of *nodD1* mutant and control samples of wild-type strain **before** normalization.

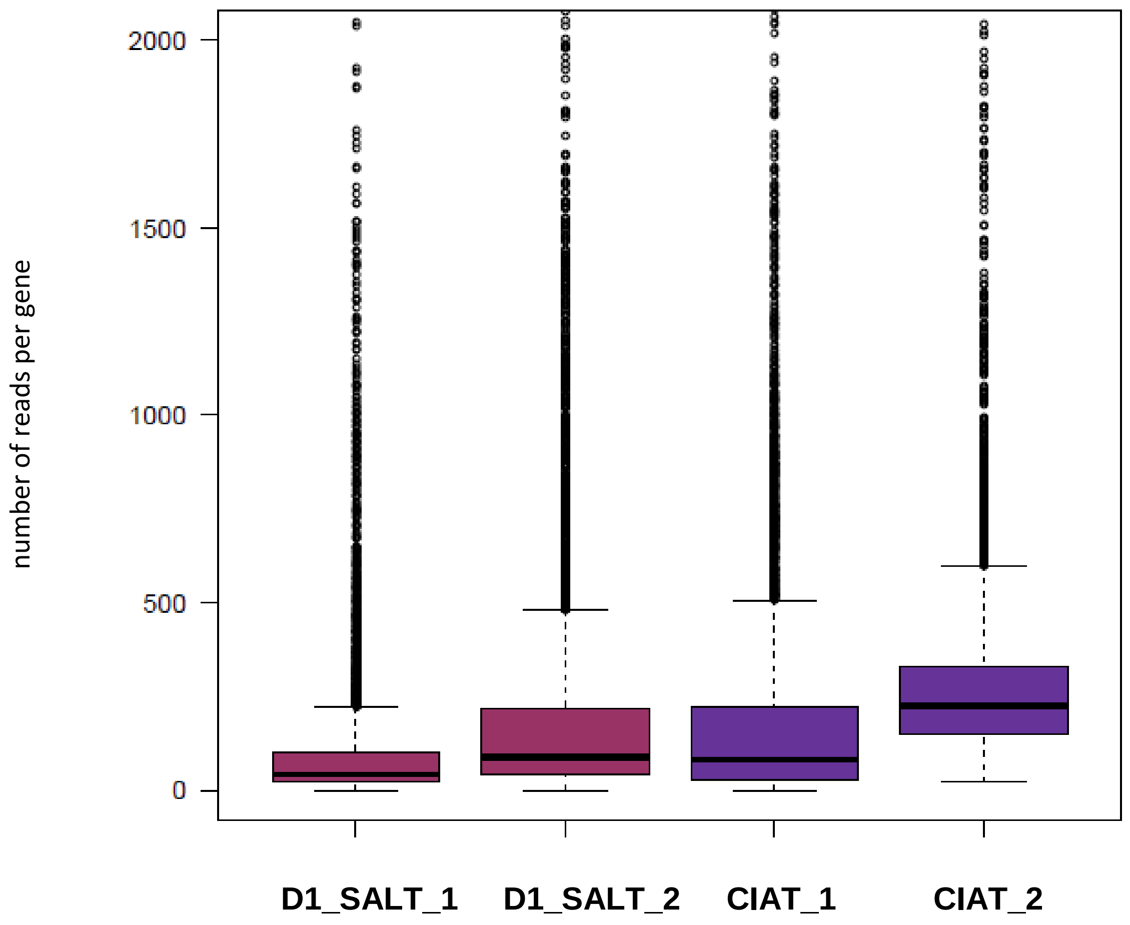

Number of reads per gene for salt samples of *nodD1* mutant and control samples of wild-type strain **after** normalization.

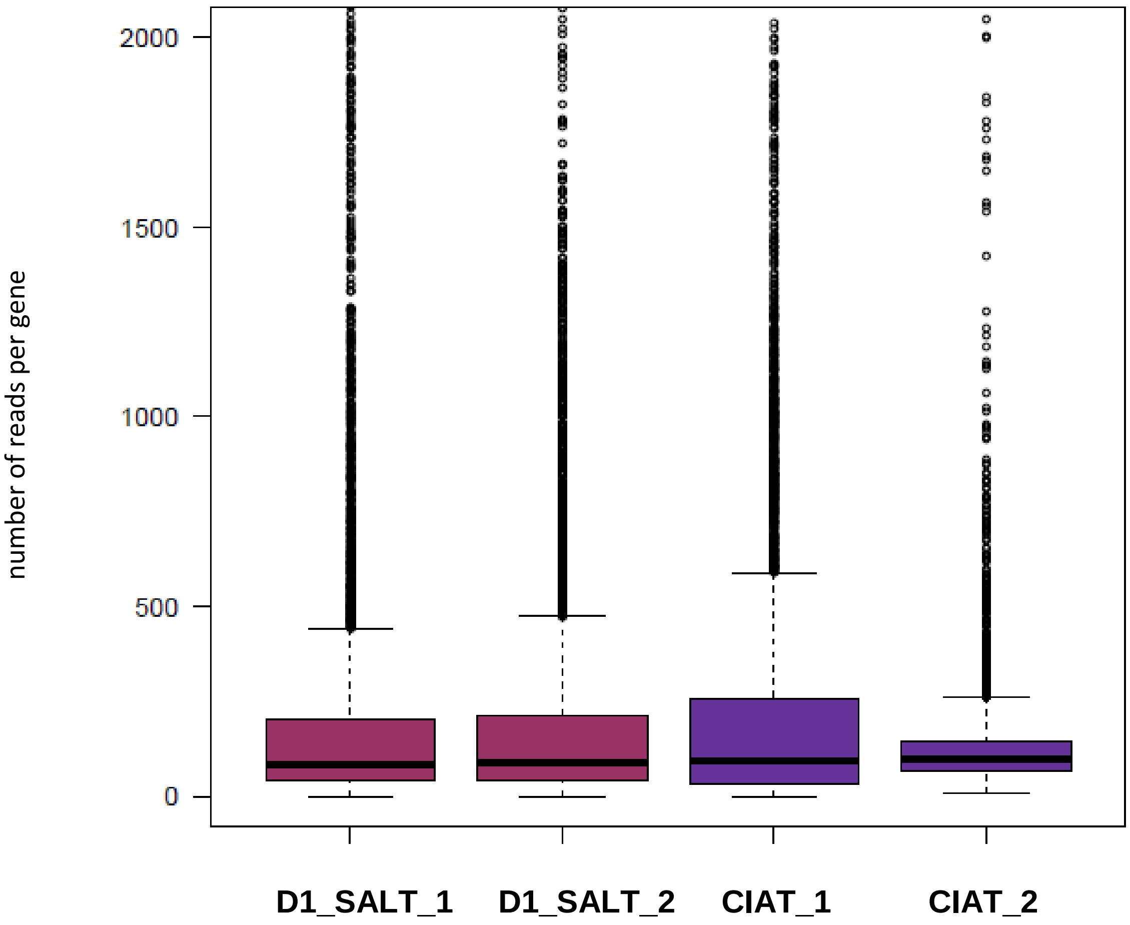

Number of reads per gene for apigenin samples of *nodD1* mutant and control samples of wild-type strain **before** normalization.

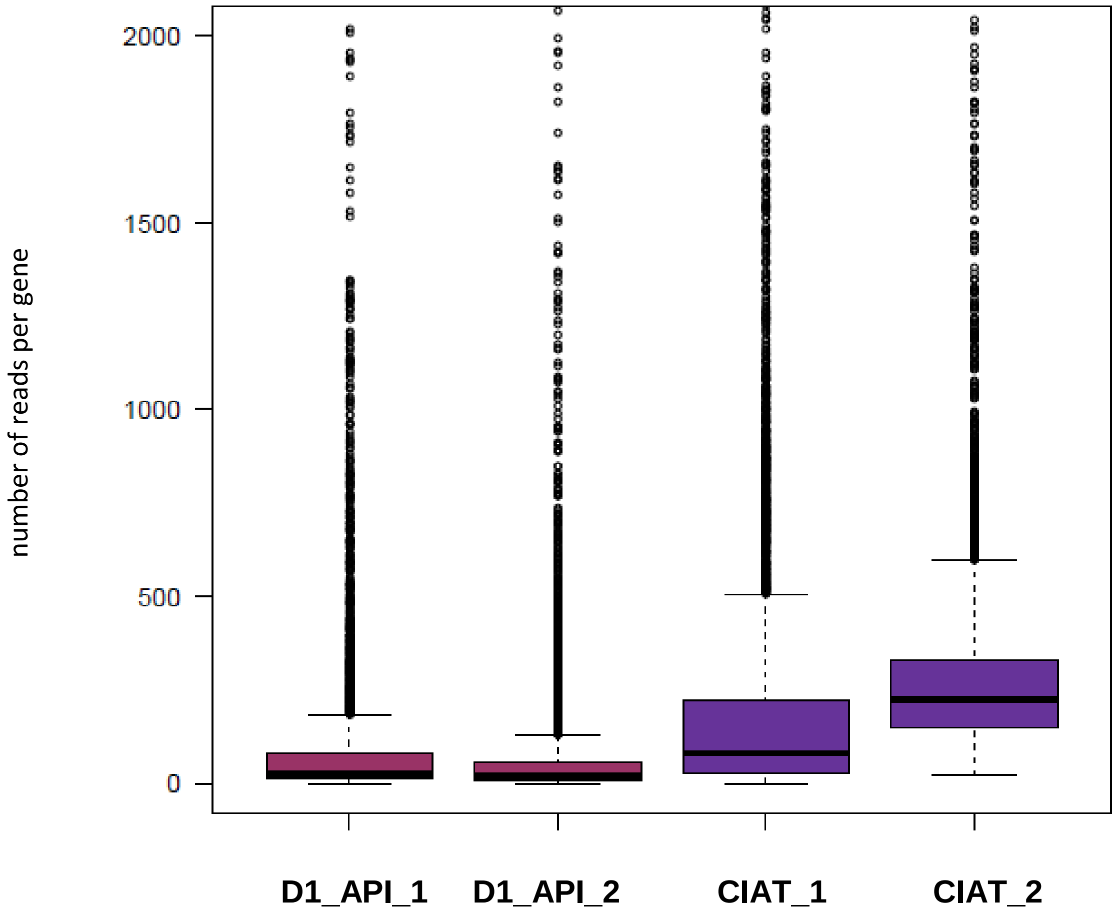

Number of reads per gene for apigenin samples of *nodD1* mutant and control samples of wild-type strain **after** normalization.

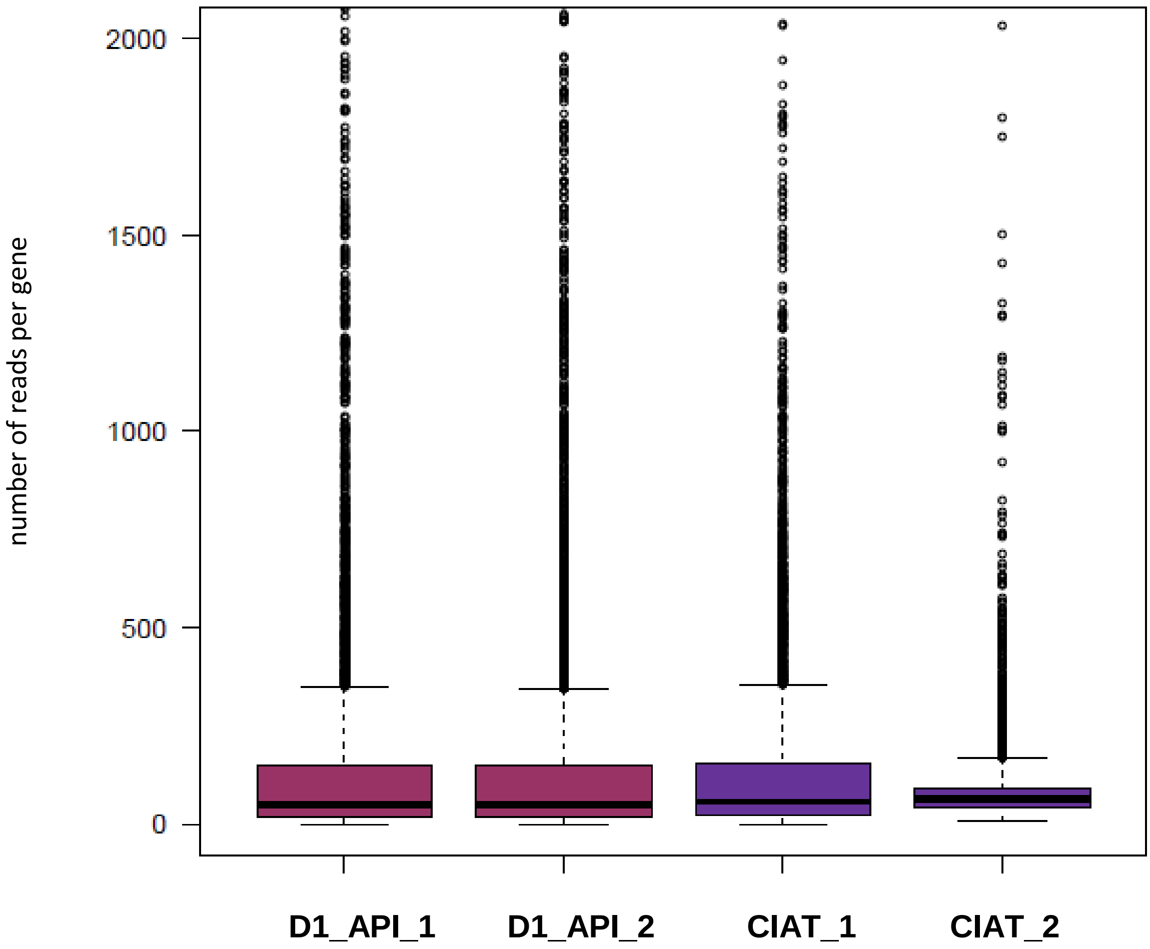

Number of reads per gene for control samples of *nodD2* mutant and wild-type strains **before** normalization.

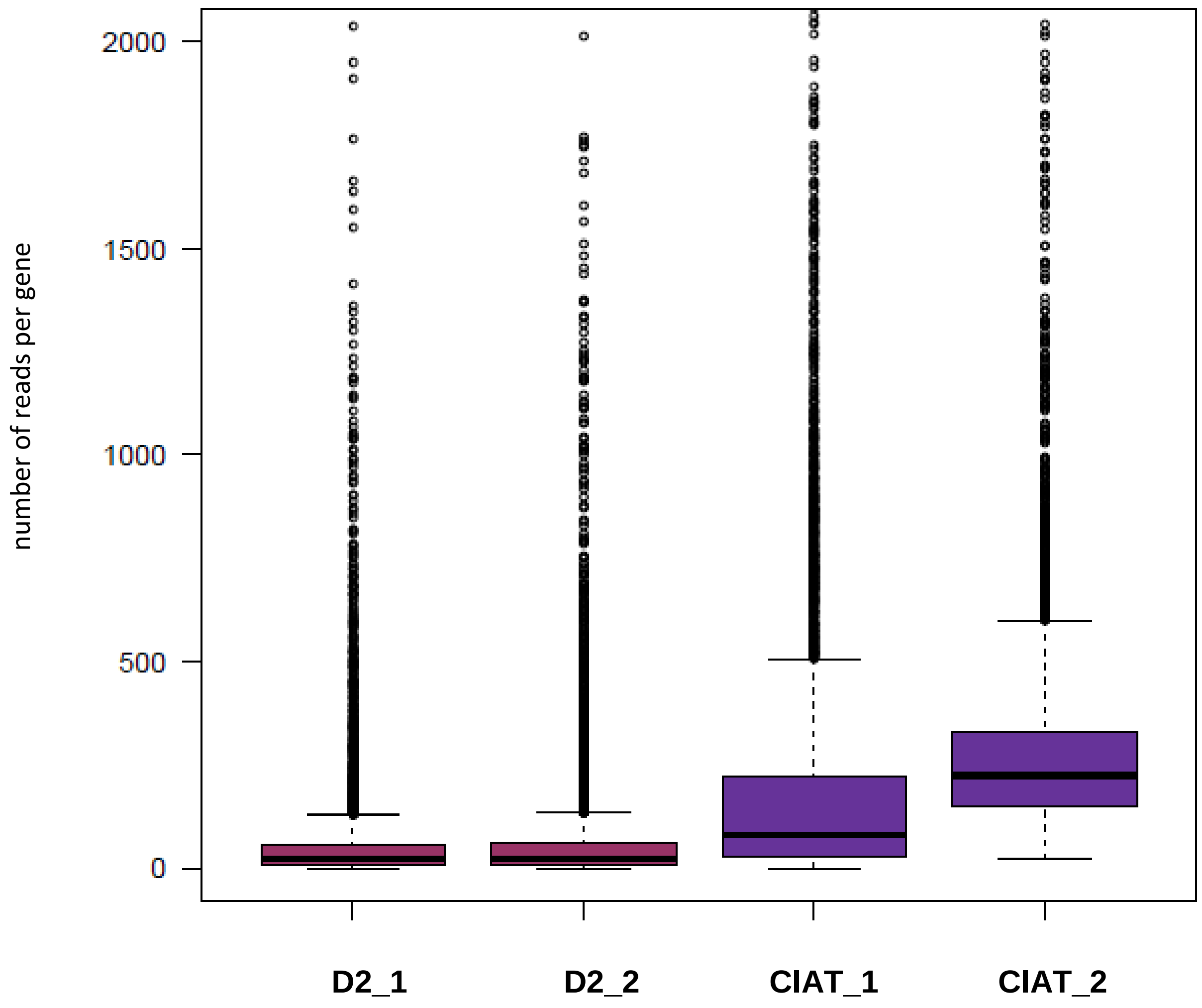

Number of reads per gene for control samples of *nodD2* mutant and wild-type strains **after** normalization.

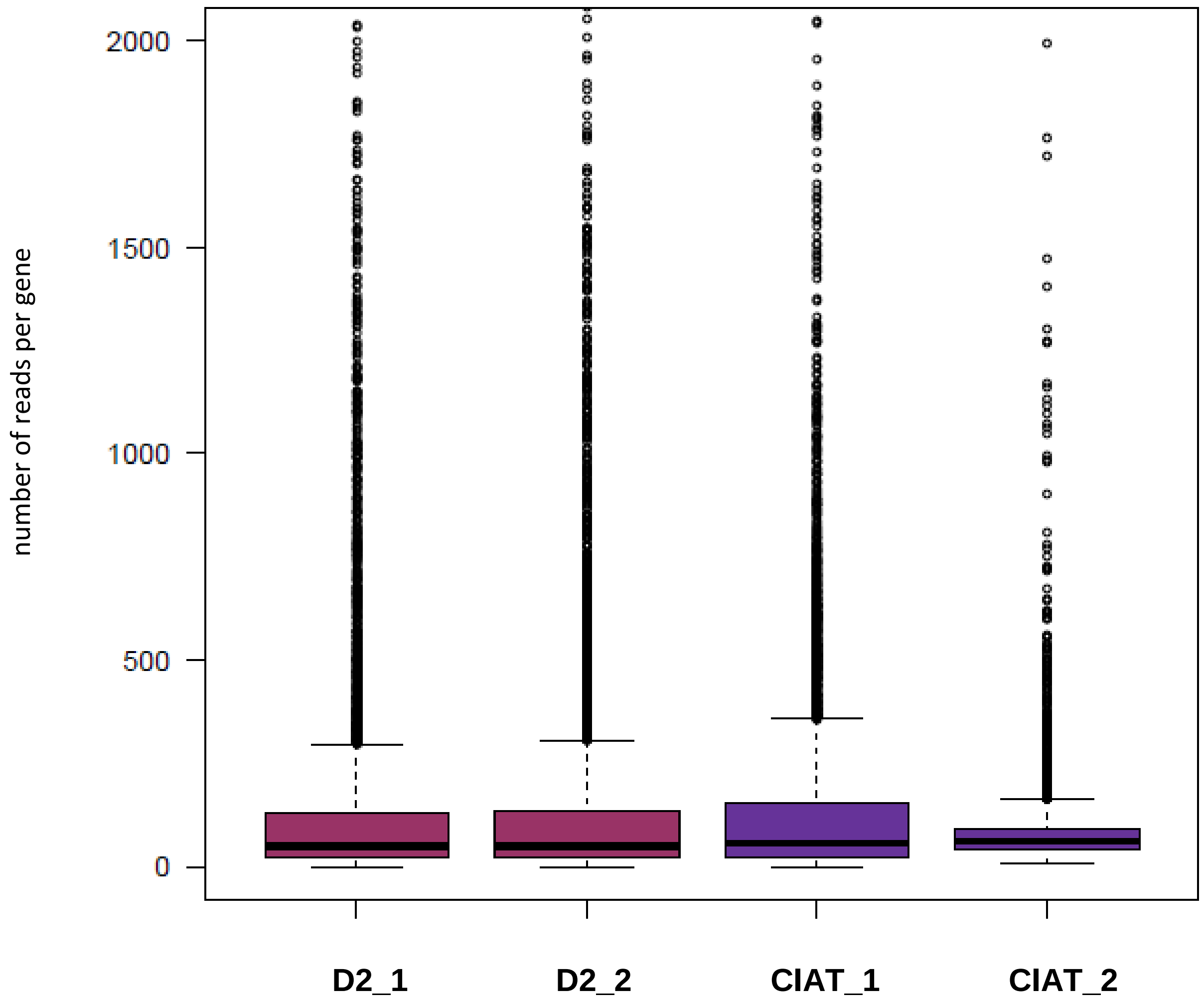

Number of reads per gene for salt samples of *nodD2* mutant and control samples of wild-type strain **before** normalization.

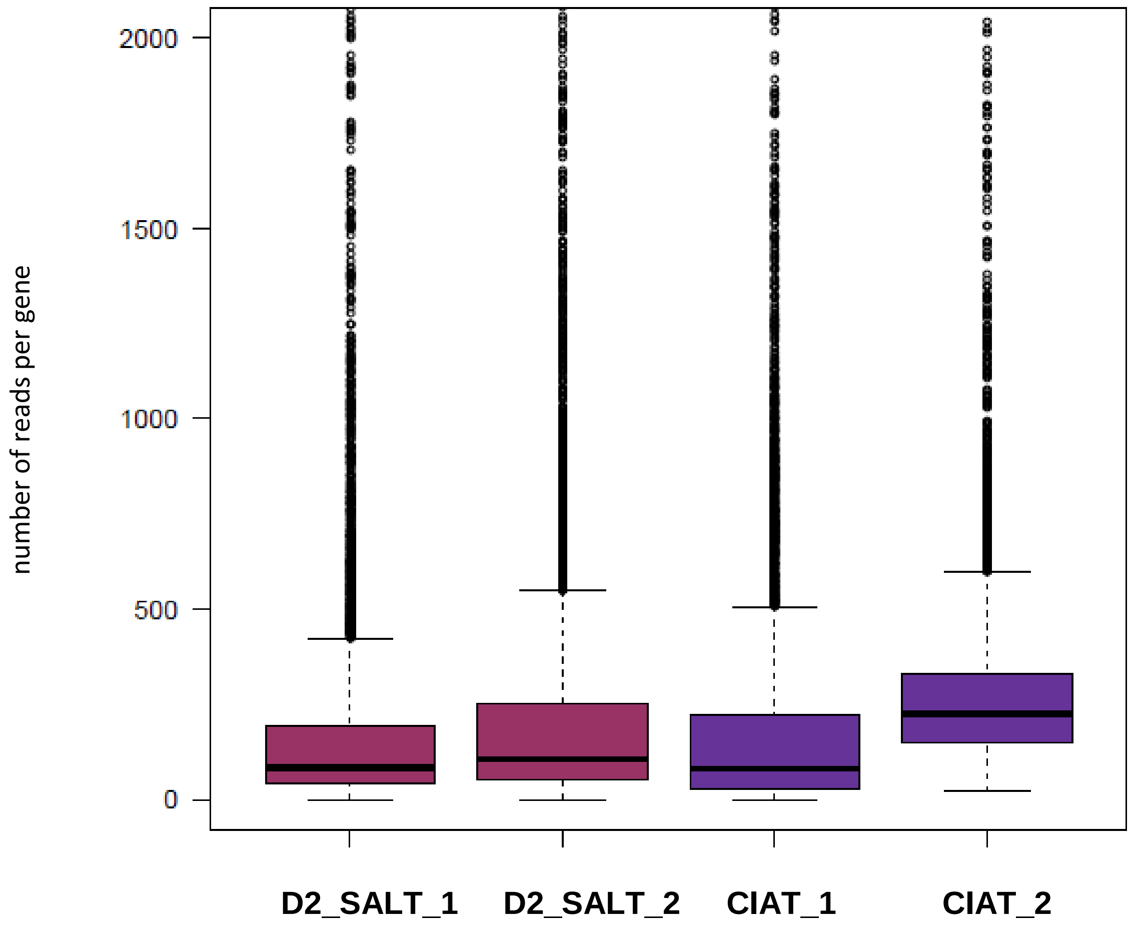

Number of reads per gene for salt samples of *nodD2* mutant and control samples of wild-type strain **after** normalization.

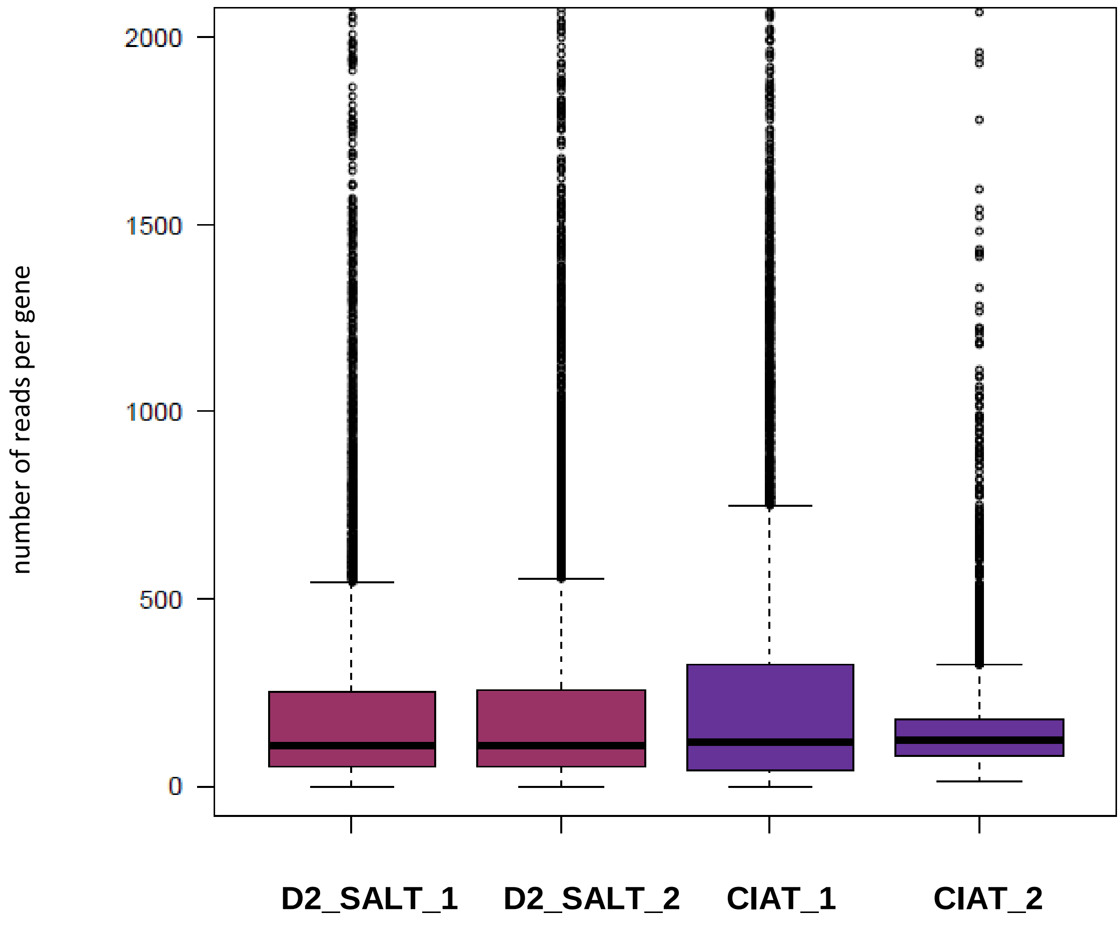

Number of reads per gene for apigenin samples of *nodD2* mutant and control samples of wild-type strain **before** normalization.

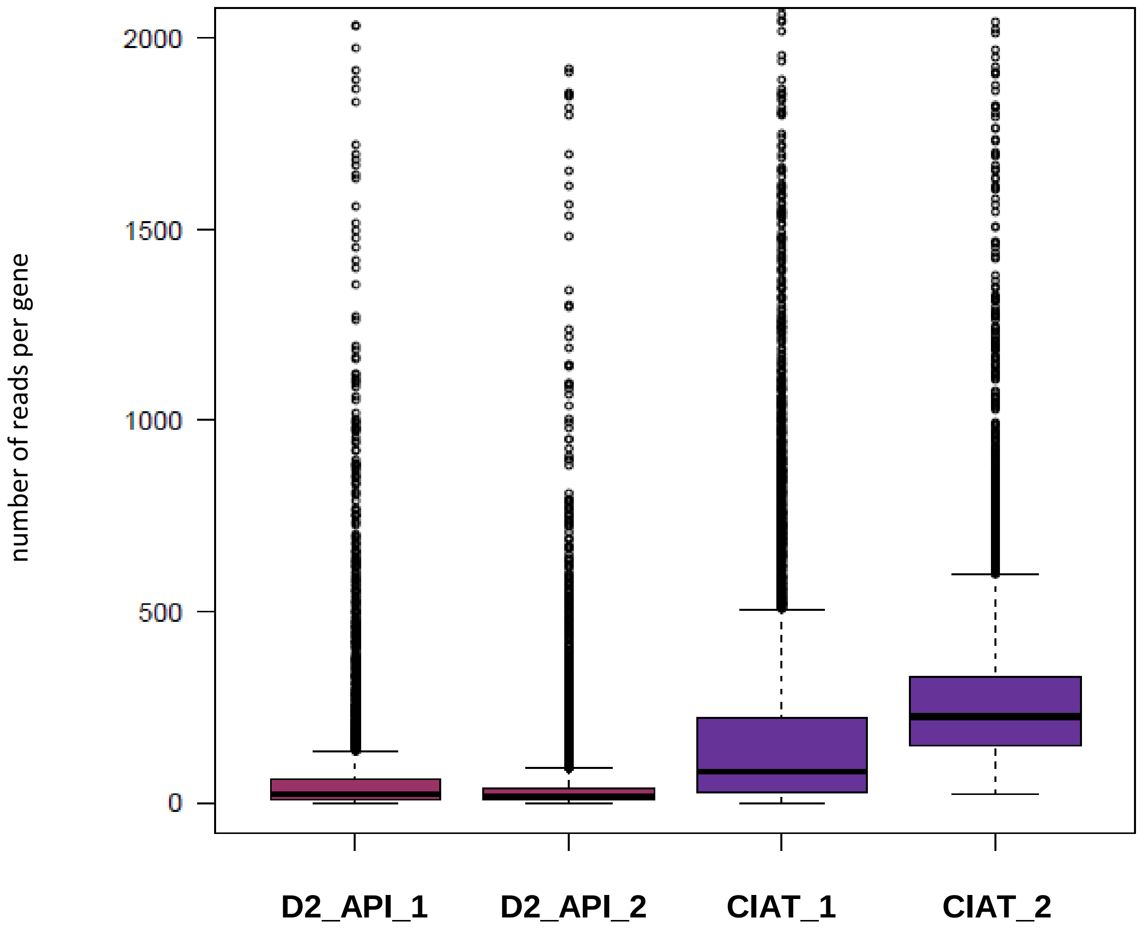

Number of reads per gene for apigenin samples of *nodD2* mutant and control samples of wild-type strain **after** normalization.

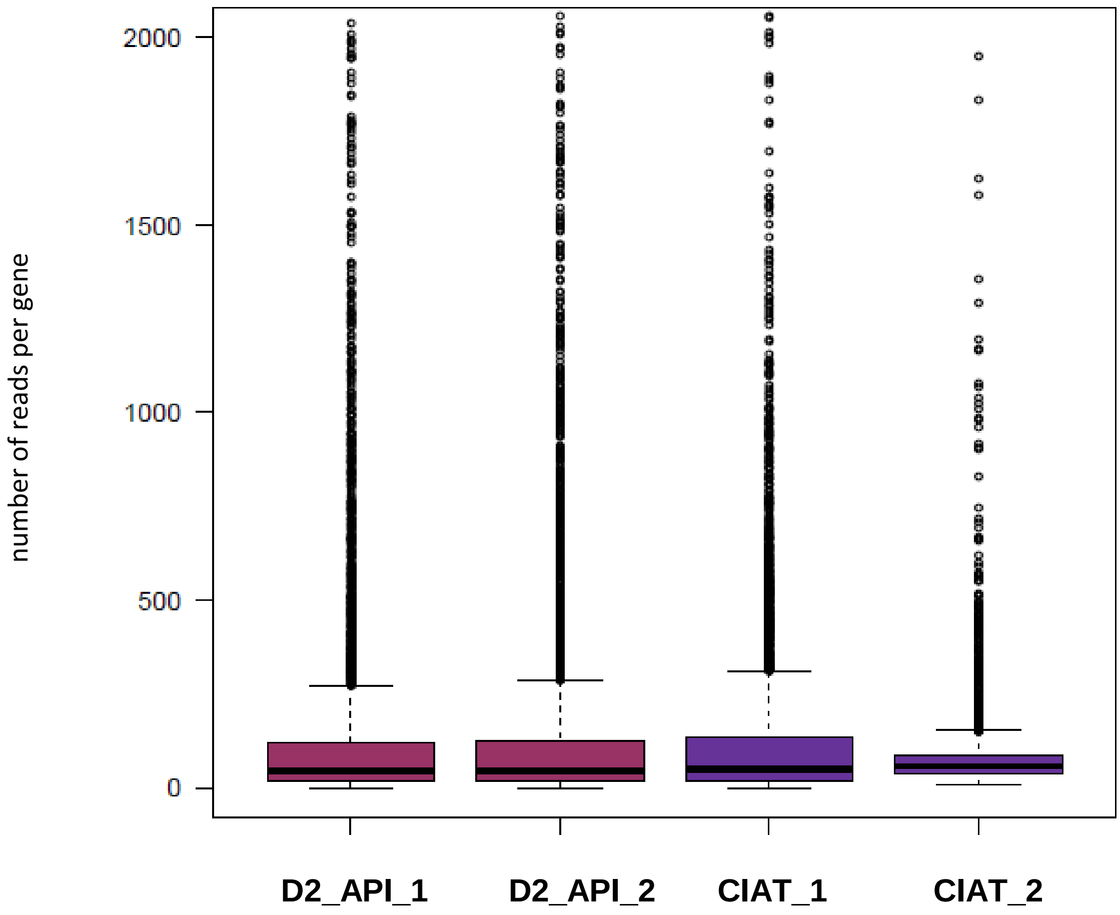

Supplementary file 5. Selected genes and primer sequences for qRT-PCR asssays.

Correlation degrees between RNA-Seq and qRT-PCR experiments.

NodD1 mutant RNA-seq

Replicon	Locus Tag	Gene name	Forward qRT-PCR primer	Reverse qRT-PCR primer
pRtrCIAT899b	RTCIAT899_PB01300	*nodA1*	TGATGCTAATGGTGTCGCGGC	ATGCCCGATCCCCAATCCCT
pRtrCIAT899b	RTCIAT899_PB01095	*nodA2*	GGGATTGTACGGGATACGCACCG	CGTGCGGAAAAACCGTCACAGG
pRtrCIAT899b	RTCIAT899_PB02710	*nodM*	TTGCAATAGCGTAGGCAAGC	TGATGTCGCCTCCGAATTTC
pRtrCIAT899b	RTCIAT899_PB00575	*y4wE*	TAGTGTTGGATGAGGCGT	CCTTCCGATAAGTTCAGGA
pRtrCIAT899b	RTCIAT899_PB01550	HP	TGAAACTTGAAGGAAGCGCG	ATAATGCCTTGCCACGTCGA
pRtrCIAT899b	RTCIAT899_PB01570	*nodD2*	AAAGCGTCTGGCAAGGGAAG	TTTTCGTCGAACAGCTTCGC

qRT-PCR			
**Gene name**	**NodD1 mutant Control**	**NodD1 mutant Apigenin**	**NodD1 mutant Salt**
nodA1	0,982820599	4,048815774	6,03142961
nodA2	0,852634892	1,411765039	4,933118694
nodM	1,328685814	2,943433431	3,99307453
y4wE	0,468136124	1,714157888	3,243384356
HP	0,69	1,73	7,43
nodD2	0,86	1,68	2,02

RNA-Seq			
**Gene name**	**NodD1 mutant Control**	**NodD1 mutant Apigenin**	**NodD1 mutant Salt**
nodA1	0,609756098	0,480769231	2,62
nodA2	0,917431193	1,41	4,14
nodM	0,598802395	0,657894737	1,68
y4wE	0,390625	0,340136054	1,88
HP	1,06	1,36	7,72
nodD2	0,53	0,657894737	1,03

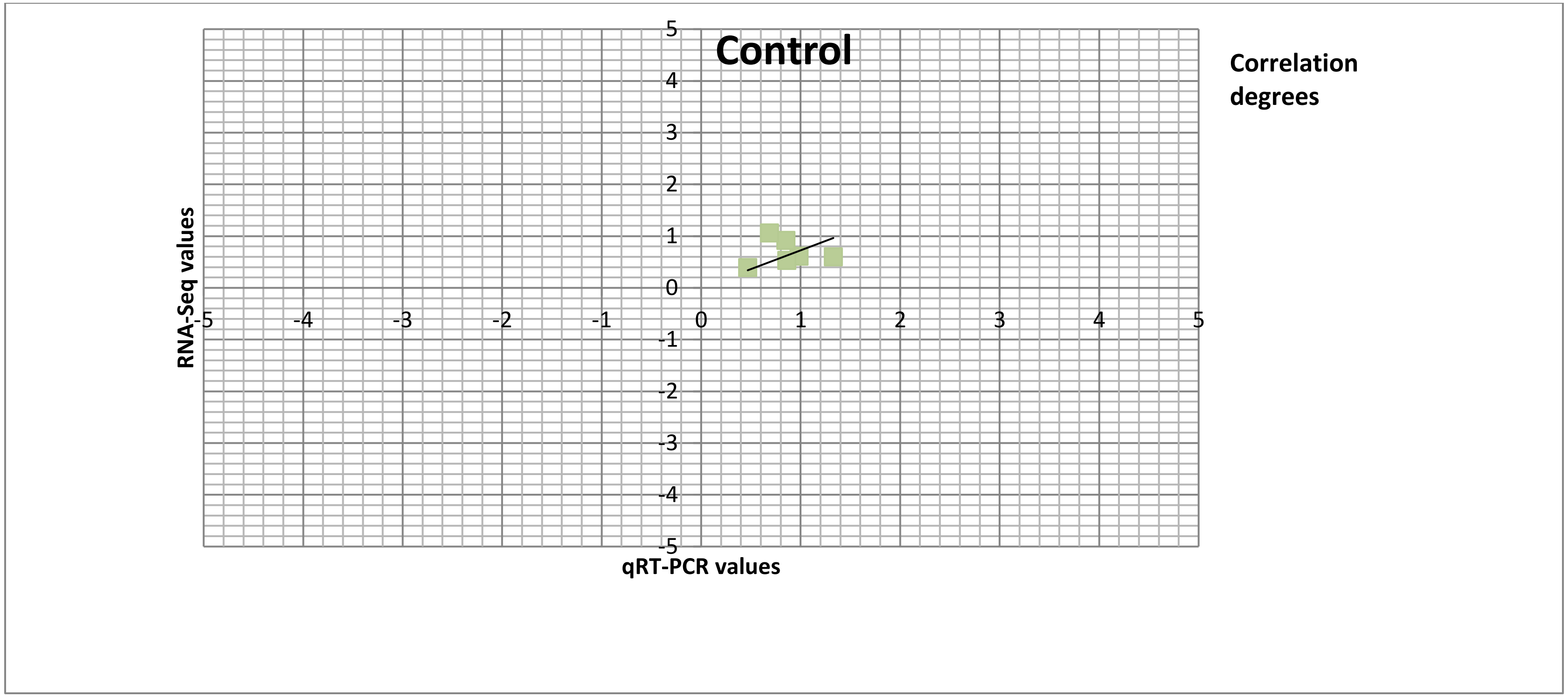
Control
Correlation degrees
RNA-Seq values
qRT-PCR values
5
4
3
2
1
0
-1
-2
-3
-4
-5
-5
-4
-3
-2
-1
0
1
2
3
4
5

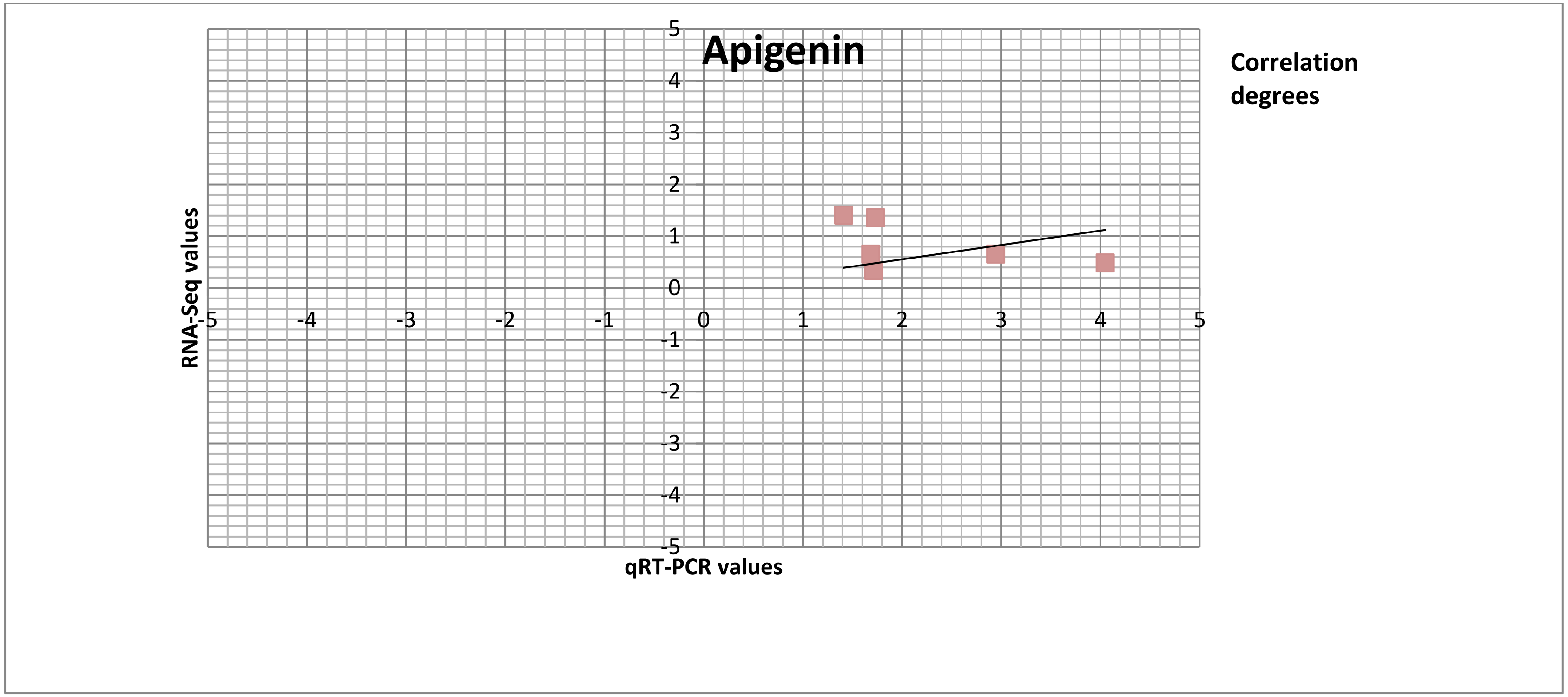
Apigenin
Correlation degrees
RNA-Seq values
qRT-PCR values
5
4
3
2
1
0
-1
-2
-3
-4
-5
-5
-4
-3
-2
-1
0
1
2
3
4
5

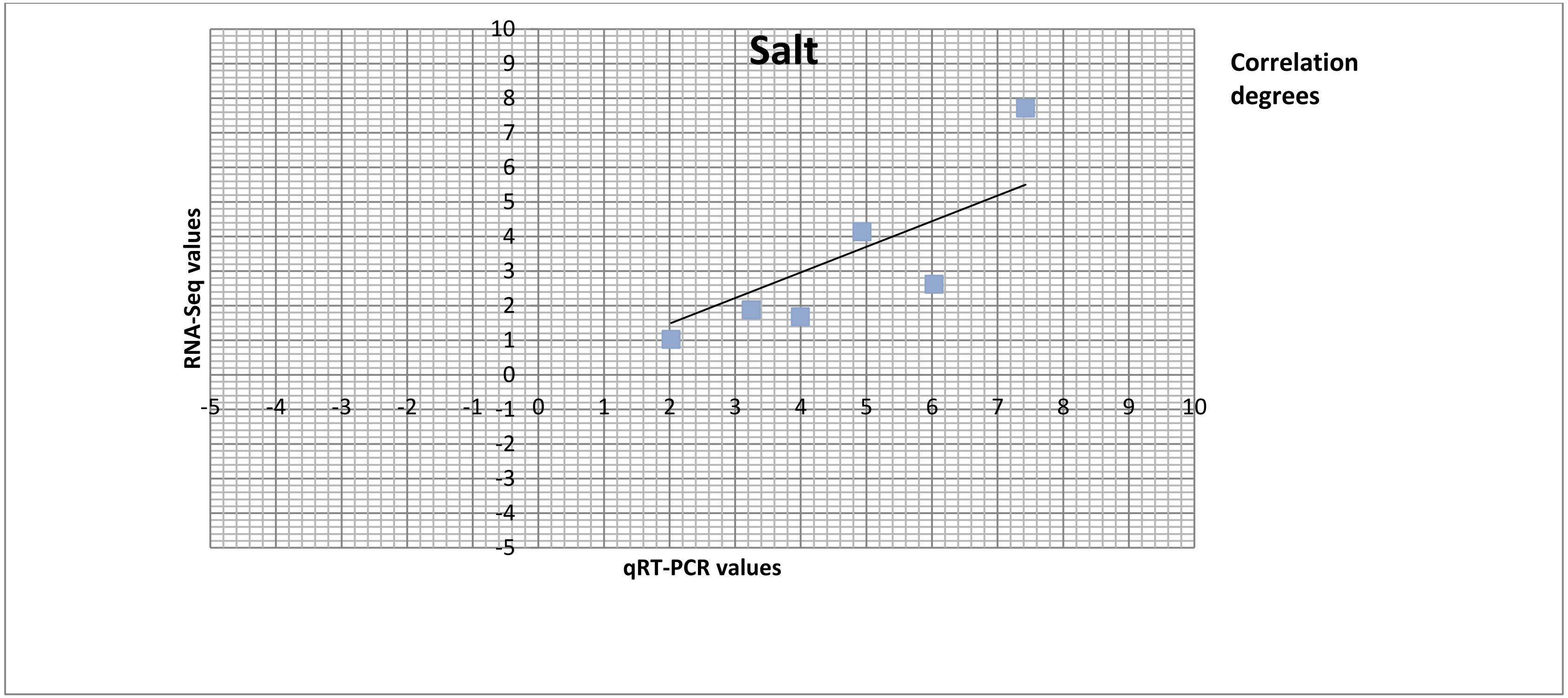
Salt
Correlation degrees
RNA-Seq values
qRT-PCR values
10
9
8
7
6
5
4
3
2
1
0
-1
-2
-3
-4
-5
-5
-4
-3
-2
-1
0
1
2
3
4
5
6
7
8
9
10

NodD2 mutant RNA-seq

Replicon	Locus Tag	Gene name	Forward	Reverse
pRtrCIAT899b	RTCIAT899_PB01300	*nodA1*	TGATGCTAATGGTGTCGCGGC	ATGCCCGATCCCCAATCCCT
pRtrCIAT899b	RTCIAT899_PB01095	*nodA2*	GGGATTGTACGGGATACGCACCG	CGTGCGGAAAAACCGTCACAGG
pRtrCIAT899b	RTCIAT899_PB02710	*nodM*	TTGCAATAGCGTAGGCAAGC	TGATGTCGCCTCCGAATTTC
pRtrCIAT899b	RTCIAT899_PB00575	*y4wE*	TAGTGTTGGATGAGGCGT	CCTTCCGATAAGTTCAGGA
pRtrCIAT899b	RTCIAT899_PB01550	HP	TGAAACTTGAAGGAAGCGCG	ATAATGCCTTGCCACGTCGA
pRtrCIAT899b	RTCIAT899_PB01570	*nodD2*	AAAGCGTCTGGCAAGGGAAG	TTTTCGTCGAACAGCTTCGC

qRT-PCR			
**Gene name**	**NodD2 mutant Control**	**NodD2 mutant Apigenin**	**NodD2 mutant Salt**
nodA1	0,860054507	17,44812372	3,830413123
nodA2	0,284697159	8,426888288	2,066945
nodM	1,650038858	10,70342044	0,623084615
y4wE	0,87206042	8,75434961	4,206149124
HP	2,68	5,15	2,02
nodD2	0,36	0,36	0,37

RNA-Seq			
**Gene name**	**NodD2 mutant Control**	**NodD2 mutant Apigenin**	**NodD2 mutant Salt**
nodA1	0,273224044	10,85	0,884955752
nodA2	0,847457627	8,29	1,08
nodM	0,662251656	2,8	1,32
y4wE	0,386100386	4,25	0,884955752
HP	1,39	4,11	1,97
nodD2	0,30	0,126262626	0,303951368

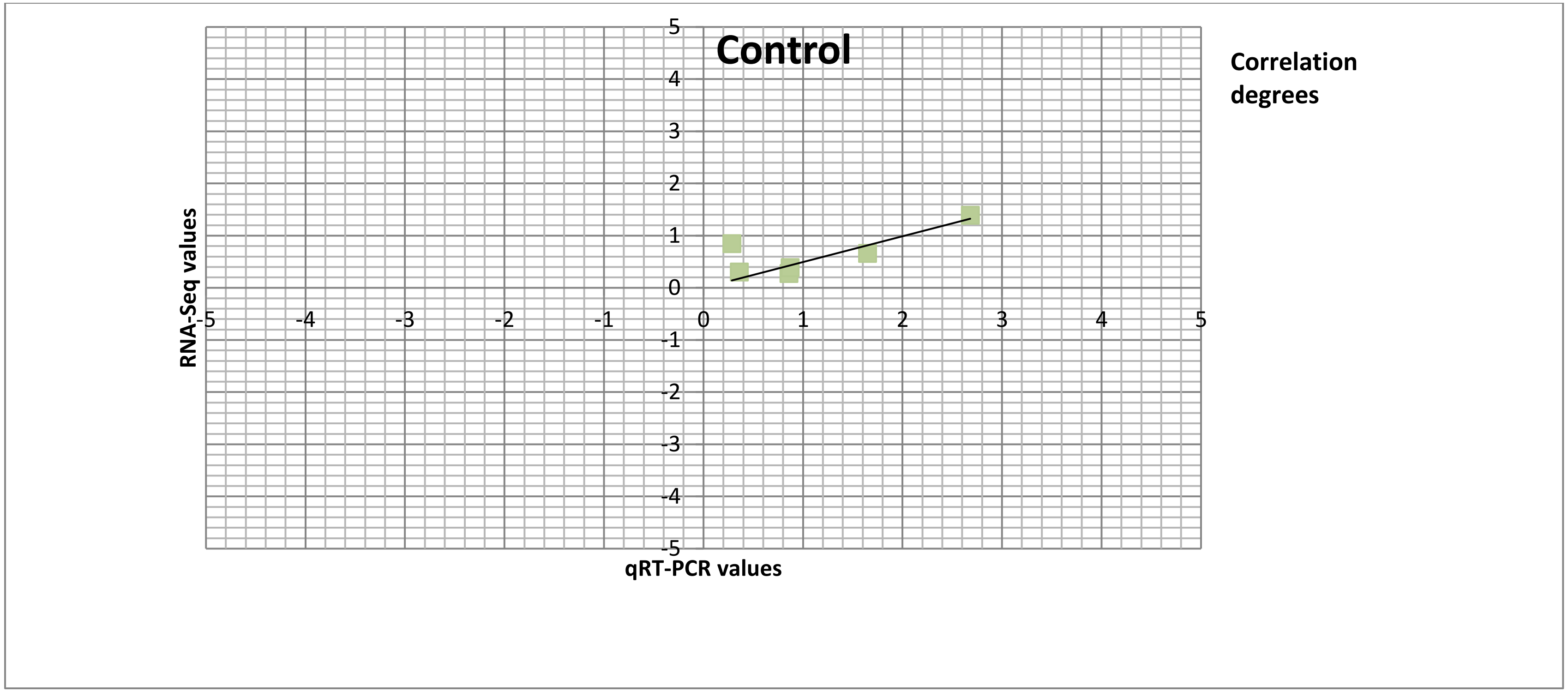
Control
Correlation
degrees
RNA-Seq values
qRT-PCR values
5
4
3
2
1
0
-1
-2
-3
-4
-5
-5
-4
-3
-2
-1
0
1
2
3
4
5

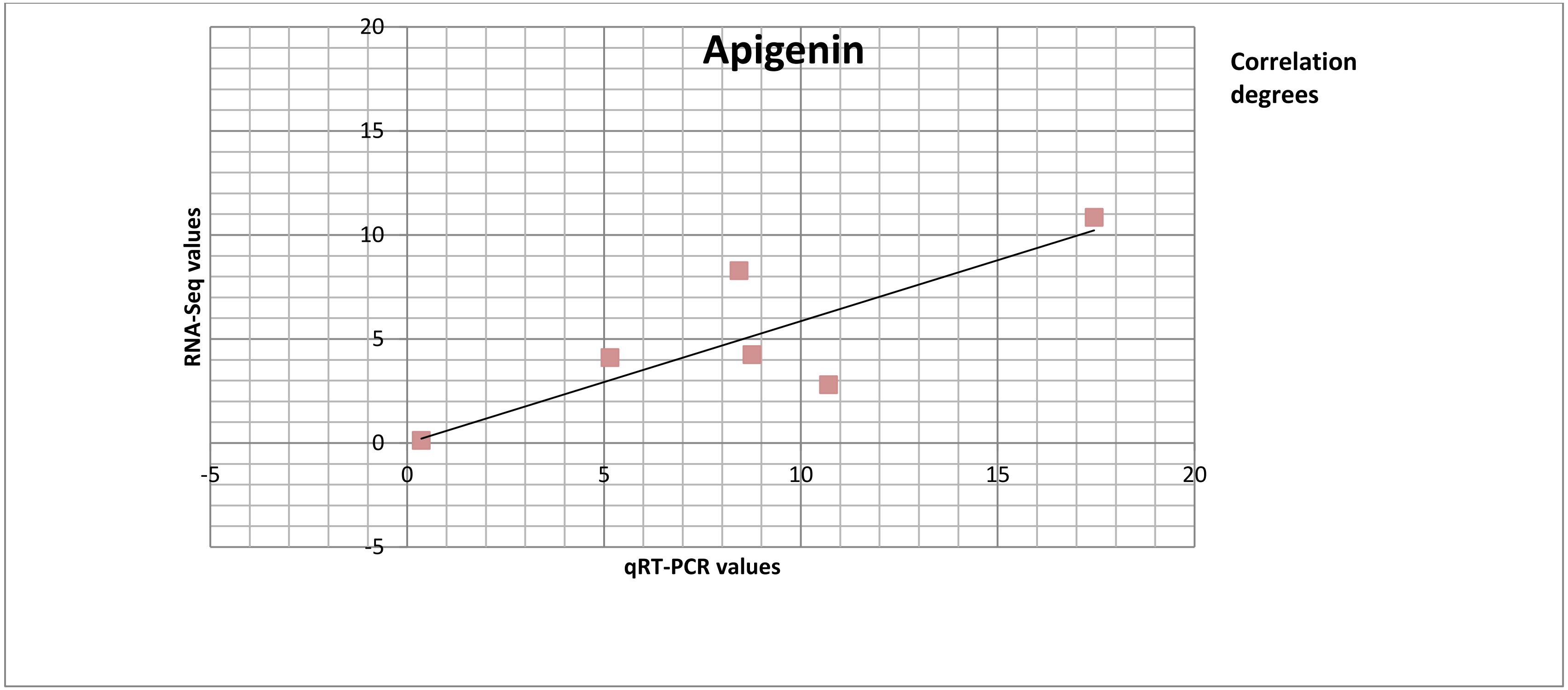

Apigenin
20
15
10
5
0
-5
-5
0
5
10
15
20
RNA-Seq values
qRT-PCR values
Correlation
degrees

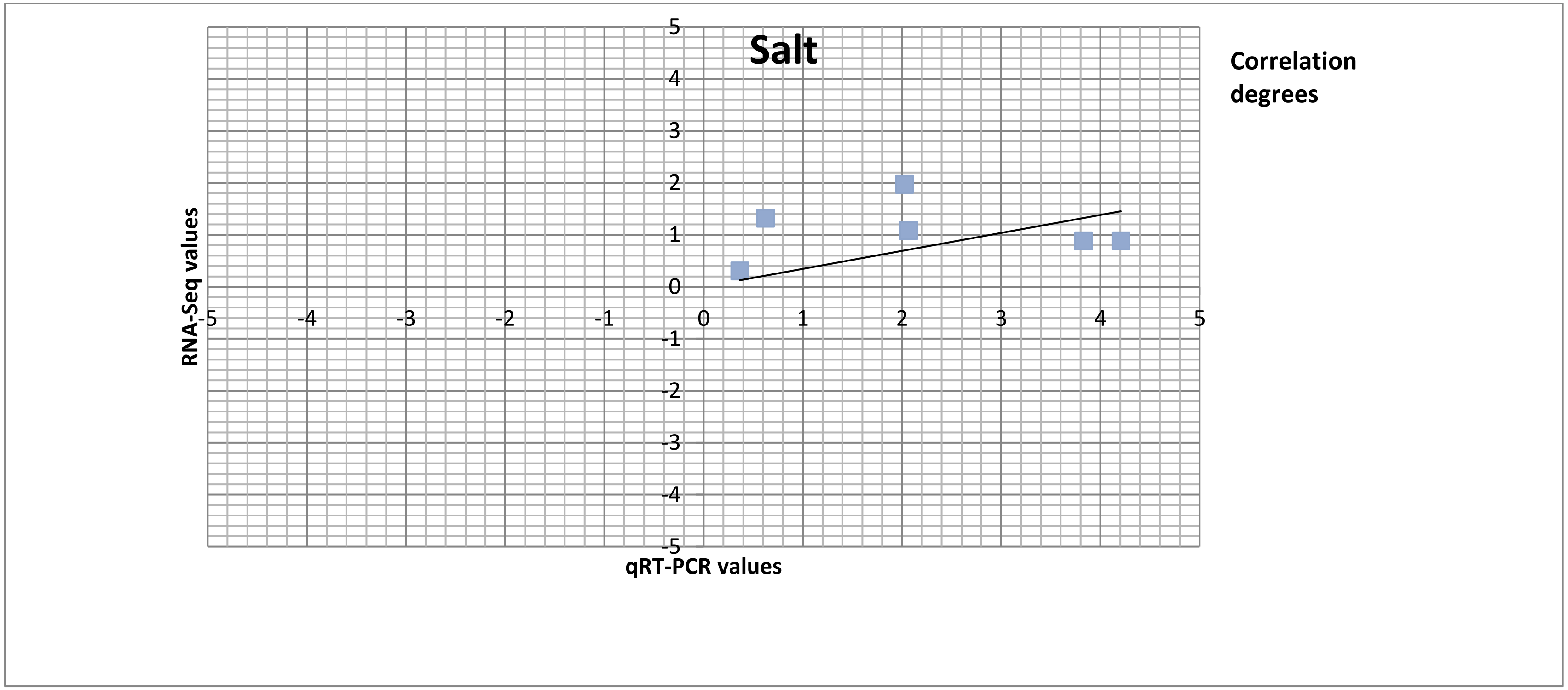
Salt
Correlation
degrees
RNA-Seq values
qRT-PCR values
5
4
3
2
1
0
-1
-2
-3
-4
-5
-5
-4
-3
-2
-1
0
1
2
3
4
5

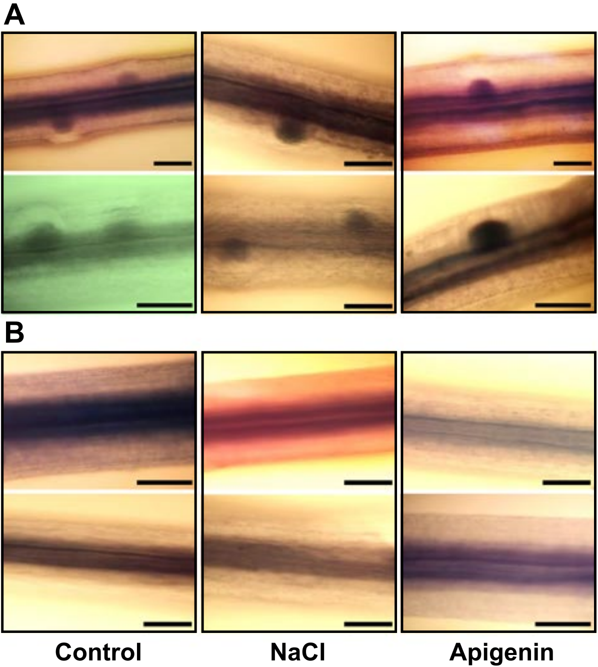
A
B
Control
NaCl
Apigenin

Supplementary file 6 legend. *Phaseolus vulgaris* roots after 10 days of grown. **A.** Pseudonodules formed after inoculation with Nod factors of *R. tropici* CIAT 899 produced in B^- medium (Control), B^- supplemented with 300 mM NaCl (NaCl) and B^- 3.7 µM apigenin (Apigenin). **B.** Absence of pseudonodules after inoculation with Nod factors of Δ*nodD1*/Δ*nodD2* strain. Nod factors production was under the same conditions observed in (**A**). Bar length = 1 mm.

Table S7. Primers employed in this study.

Name of the primer	Nucleotide sequence
nodD1.A	ATAAAGCTTCGATGAATGGGCCGTCCA
nodD1.B	TCAATCTCAGCCAGCATTGAGTGGATGC
nodD1.C	GCATCCACTCAATGCTGGCTGAGATTGA
nodD1.D	AAAGGATCCGCCGATGTACTCGTCTGCTA
nodD2.A	ATAAAGCTTGTAGGCCATAATGTCCAGA
nodD2.B	ACACATCGAAGGACGATCAAGACCCTTG
nodD2.C	CAAGGGTCTTGATCGTCCTTCGATGTGT
nodD2.D	AAAGGATCCGCGGCTTTATACTCACCA

Original TLC 1

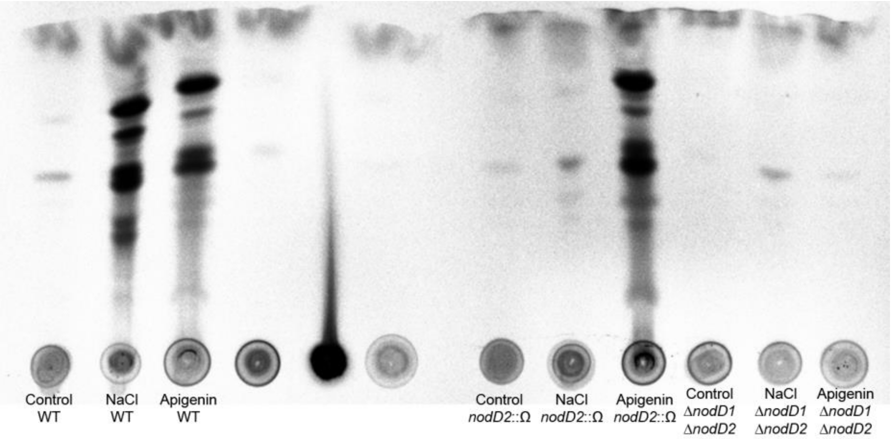

Original TLC 2

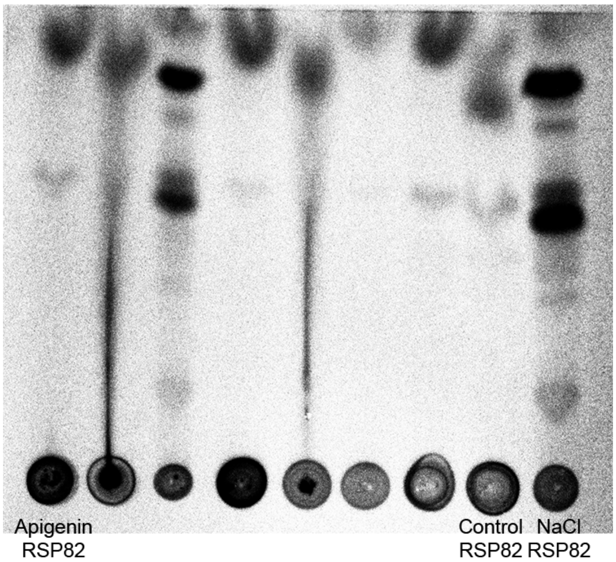

Supplement: Supplementary files 1-7 [file srep46712-s1.pdf]
